# Metabolic landscape of the healthy pancreas and pancreatic tumor microenvironment

**DOI:** 10.1172/jci.insight.180114

**Published:** 2024-08-13

**Authors:** Monica E. Bonilla, Megan D. Radyk, Matthew D. Perricone, Ahmed M. Elhossiny, Alexis C. Harold, Paola I. Medina-Cabrera, Padma Kadiyala, Jiaqi Shi, Timothy L. Frankel, Eileen S. Carpenter, Michael D. Green, Cristina Mitrea, Costas A. Lyssiotis, Marina Pasca di Magliano

**Affiliations:** 1 Program in Cancer Biology;; 2 Department of Molecular and Integrative Physiology;; 3 Program in Immunology;; 4 Department of Computational Medicine and Bioinformatics;; 5 Rogel Cancer Center;; 6 Department of Pathology;; 7 Department of Surgery;; 8 Department of Internal Medicine, Division of Gastroenterology and Hepatology;; 9 Department of Radiation Oncology; and; 10 Department of Microbiology and Immunology, University of Michigan, Ann Arbor, Michigan, USA.; 11 Department of Radiation Oncology, Veterans Affairs Ann Arbor Healthcare System, Ann Arbor, Michigan, USA.; 12 Department of Cell and Developmental Biology, University of Michigan, Ann Arbor, Michigan, USA.

**Keywords:** Oncology, Bioinformatics, Cancer, Macrophages

## Abstract

Pancreatic cancer, one of the deadliest human malignancies, is characterized by a fibro-inflammatory tumor microenvironment and wide array of metabolic alterations. To comprehensively map metabolism in a cell type–specific manner, we harnessed a unique single-cell RNA-sequencing dataset of normal human pancreata. This was compared with human pancreatic cancer samples using a computational pipeline optimized for this study. In the cancer cells we observed enhanced biosynthetic programs. We identified downregulation of mitochondrial programs in several immune populations, relative to their normal counterparts in healthy pancreas. Although granulocytes, B cells, and CD8^+^ T cells all downregulated oxidative phosphorylation, the mechanisms by which this occurred were cell type specific. In fact, the expression pattern of the electron transport chain complexes was sufficient to identify immune cell types without the use of lineage markers. We also observed changes in tumor-associated macrophage (TAM) lipid metabolism, with increased expression of enzymes mediating unsaturated fatty acid synthesis and upregulation in cholesterol export. Concurrently, cancer cells exhibited upregulation of lipid/cholesterol receptor import. We thus identified a potential crosstalk whereby TAMs provide cholesterol to cancer cells. We suggest that this may be a new mechanism boosting cancer cell growth and a therapeutic target in the future.

## Abstract 2

Using computational approaches, we discovered metabolic adaptations of cancer cells and components of the pancreatic tumor microenvironment, including dysregulation of fatty acid and cholesterol synthesis.

## Graphical abstract

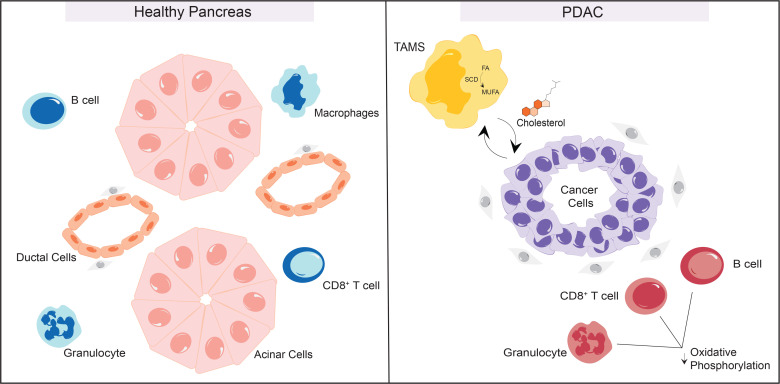

## Introduction

Pancreatic ductal adenocarcinoma (PDA) is a lethal disease with a current 5-year survival rate of 13% (1). Its poor prognosis can be attributed to a lack of early detection methods and a paucity of effective therapeutic options. Indeed, at diagnosis most patients present with locally advanced or metastatic disease that is refractory to chemotherapy, radiotherapy, and immunotherapy (2). At the genetic level, pancreatic cancer is almost invariably associated with mutations in Kras, together with common loss of tumor suppressor genes such as *TP53*, the *INK4A* locus, and *SMAD4* (3). A hallmark of pancreatic cancer is the extensive tumor microenvironment (TME), a conglomerate of fibroblasts, immune cells, and noncellular components of the extracellular matrix, that is hypovascularized and extremely nutrient deprived (3, 4). Within the TME, fibroblasts and immune cells actively support cancer cells, allowing them to persist and grow even in the absence of adequate vascularization (5). PDA cells scavenge nutrients to circumvent limited supply, sourcing nonclassical nutrients from their environment through expression of high-avidity nutrient transporters, bulk engulfment, and crosstalk with other cell types (6–11). Competition for nutrients promotes immune cell dysfunction (12). Metabolic restrictions imposed on T cells have been shown to decrease proliferation and cytotoxic effector functions, which dampen antitumor responses (13). Tumor-associated macrophages (TAMs), the most prevalent immune cell type in pancreatic cancer, exert tumor-promoting and immunosuppressive functions through multiple parallel mechanisms, including expression of immune checkpoint ligands, production of tumor-supporting growth factors, and production of immunosuppressive cytokines (14). Among the mechanisms through which TAMs promote immunosuppression is depletion of nutrients that are essential for T cell proliferation/activation, such as arginine, through expression of the enzyme arginase (15–17); arginine is one of the most depleted nutrients in the pancreatic TME (18). In addition to dampening immune responses, arginine depletion directly benefits cancer cells (19). In another role, TAMs can also provide cancer cells with pyrimidines, a building block for DNA required for proliferation, in the process also conferring resistance to pyrimidine nucleoside analog chemotherapeutics such as gemcitabine (20). However, a comprehensive map of metabolic coadaptations across cell types in human pancreatic cancer has so far been missing.

Until recently, one of the challenges with generating data from normal pancreas was the lack of single-cell–level gene expression data. The latter is explained by the absence of clinical indications for sampling normal pancreas in healthy individuals and by the rapid degradation of pancreas tissue through autodigestion after death. As a result, most studies have used adjacent normal samples as controls; this approach has significant limitations, as the adjacent normal pancreas presents with morphologic and inflammatory changes that lead to gene expression alterations (21). Through a unique partnership with Gift of Life Michigan, an organ donation organization, we have obtained pancreata from healthy individuals of varied ages, sexes, and races and performed single-cell RNA sequencing (scRNA-Seq) (21). The availability of “truly normal” pancreas has given us an opportunity to define metabolic alterations, at the transcriptional level, between the normal pancreas and pancreatic cancer on a cell type by cell type basis. Notably, we showed that normal human pancreas frequently harbors premalignant lesions, an aspect that is not mimicked by mouse models and supports the need to perform this type of analysis in human samples.

To investigate both (i) cell type–specific metabolic changes and (ii) coordinated changes between cell types that promote cooperative metabolism in the pancreatic microenvironment, we leveraged scRNA-Seq data from normal human pancreata and human pancreatic tumors (21, 22). On these data, we performed differential gene expression (DGE) analysis, gene set enrichment analysis (GSEA), and transcription factor activity analysis. In addition, we also assessed for cooperative metabolic crosstalk pathways that were differentially regulated in cancer relative to normal tissue. This collective analysis revealed prominent changes in amino acid and vitamin metabolism in the epithelial compartment that recapitulated previous findings (11, 23, 24). We also discovered alterations that were not previously described to our knowledge. In the immune compartments, multiple tumor-associated immune cell subtypes had decreased expression of the oxidative phosphorylation signature compared with their counterparts in the healthy pancreas. Intriguingly, the specific gene expression signatures were cell type specific.

We then investigated reciprocal metabolic interactions between cancer cells and components of the microenvironment. Notably, TAMs were found to upregulate the cholesterol exporter ATP binding cassette subfamily G member 1 (*ABCG1*), while pancreatic cancer cells differentially increased expression of the cognate receptor low-density lipoprotein receptor (*LDLR*). This interaction was validated through immunofluorescence staining of human tissue, suggesting a previously undescribed metabolic alteration in pancreatic cancer cells that allows the prioritization of cholesterol scavenging relative to biosynthesis. Overall, our study provides an atlas of metabolic alterations engendered in pancreatic cancer across multiple cellular compartments, complementing previous work mapping cellular interactions driven by receptor-ligand expression, described in our previous studies (21, 22), which may promote cancer cell growth and maintenance of an immunosuppressive microenvironment resistant to existing therapeutic strategies.

## Results

### Single-cell atlas from healthy human pancreata and pancreatic cancer samples reveals metabolic alterations in several compartments of the TME.

**Figure 1:**
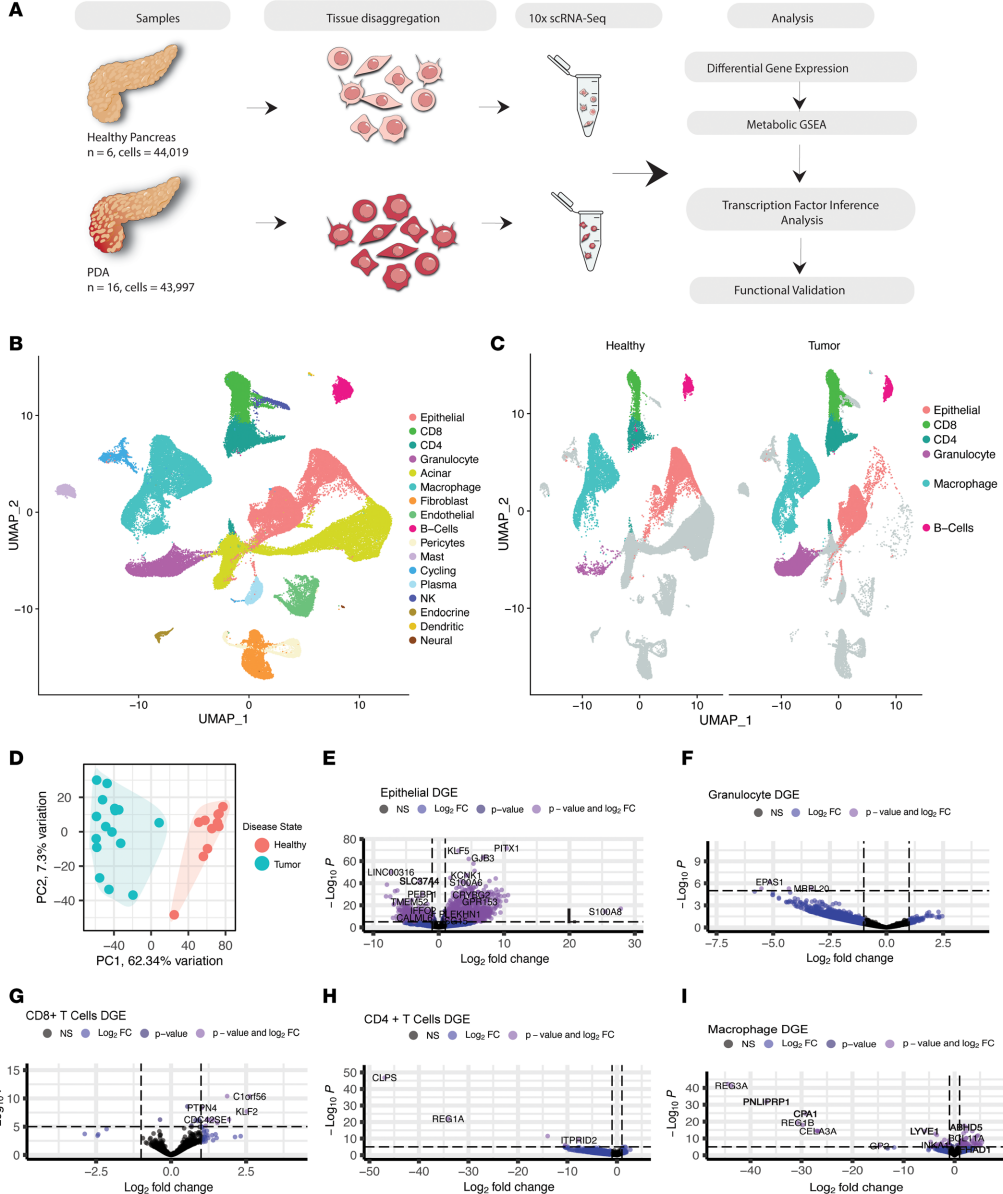
Data composition and workflow. (**A**) Schematic of single-cell sequencing performed on 6 healthy pancreata procured from a collaboration with the Gift of Life Michigan, a center for organ and tissue procurement, and 16 pancreatic cancer samples: 10 from surgical resections and 6 from fine needle biopsies at the University of Michigan. Followed by analysis workflow. (**B**) Uniform manifold approximation and projection (UMAP) visualization of all identified cell types present in the pancreatic microenvironment. (**C**) UMAP visualization of cell types that demonstrated significant metabolic alterations in the pancreatic cancer samples compared with healthy human pancreas tissue when GSEA was performed with metabolic gene sets. (**D**) Principal component analysis (PCA) plot of healthy human pancreata samples and PDA samples. (**E**–**I**) Volcano plots of DGE by cell type. Genes that are significantly up- (top right) and downregulated (top left) in tumor versus heathy and the gene symbols are included for representative differentially expressed genes.

To query metabolic reprogramming across the pancreatic TME, we leveraged datasets previously published by our laboratory, including pancreatic cancer (*n* = 16, across disease stages) and normal pancreas (*n* = 6) (21, 22). Using DGE analysis, GSEA, and transcription factor inference analysis, we sought to understand metabolic alterations in malignant and nonmalignant cells in the pancreas (Figure 1A). The data, visualized using uniform manifold approximation and projection (UMAP), included 44,019 cells from healthy pancreata and 43,997 cells from pancreatic cancer samples (Figure 1, B and C). Healthy and tumor samples readily segregated based on gene expression profiles (Figure 1D).

In the healthy exocrine pancreas, the epithelial compartment is composed of acinar, ductal, and endocrine cells. While both acinar and ductal cells can give rise to pancreatic cancer in mouse models, cancer cells are more transcriptionally similar to ductal cells. Conversely, acinar cells have a specific transcriptional profile characterized by a prevalence of genes encoding digestive enzymes (25, 26). To compare tumor and healthy tissue, we excluded acinar cells from our analysis and focused primarily on ductal and malignant cells, hereafter referred to as nonacinar epithelial cells. As expected, acinar cells were mostly detected in healthy tissue, while nonacinar epithelial cells, fibroblasts, and multiple immune compartments were present in both healthy and malignant tissue, though immune cells were more abundant in tumors (Supplemental Figure 1A; supplemental material available online with this article; https://doi.org/10.1172/jci.insight.180114DS1). A few populations, namely endocrine, dendritic, and neural cells, had limited representation with only hundreds of cells. Due to low statistical power, these cell types were interrogated only in a subset of our analyses.

DGE analysis allows for the investigation of differentially regulated genes that drive multiple biological processes, including metabolism, on a per–cell type basis. Application of DGE analysis on tumor samples relative to normal revealed differential upregulation of 4,977 genes and downregulation of 4,104 genes in nonacinar epithelial cells, which include normal ductal cells and cancer cells (Figure 1E). Granulocytes from tumors exhibited differential upregulation of 18 genes and downregulation of 812 genes (Figure 1F). Among lymphocyte populations, CD8^+^ T cells from pancreatic tumors exhibited significant differential upregulation of 287 genes and downregulation of 89 genes; in CD4^+^ T cells, we observed 121 upregulated and 6,530 downregulated genes, respectively (Figure 1, G and H). Macrophages upregulated 993 genes and downregulated 881 genes (Figure 1I). We also analyzed several less abundant types of cells; however, due to limited cell number, the DGE analysis was less informative (Supplemental Figure 1, C–H).

We next performed transcription factor inference analysis to ascertain master regulatory networks in the healthy human pancreas and PDA samples (Supplemental Data 1 and 2). Further, we performed analysis to distinguish transcription factors that had the highest log fold-change in PDA samples compared with healthy tissue (Supplemental Data 3). We used the single-cell regulatory network inference and clustering (SCENIC) package in R to infer putative regulon activity. Transcription factor motif enrichment analysis enables the identification of gene targets regulated by a transcription factor — these comprise a regulon, and AUCell assigns a corresponding regulon activity score to cells (27). Collectively, these analyses revealed changes in regulon activity for corresponding transcription factors by cell type and disease status. Rather than solely rely on the expression of transcription factors, this package measures the expression of target genes of each transcription factor, thus providing an activity measure. Another advantage of this method is that it avoids “dropout,” a limitation of scRNA-Seq whereby lowly expressed genes (as transcription factors often are) might show incorrectly as not expressed.

In epithelial cells, we observed increased activity for transcription factors that positively regulate cell proliferation (*GRHL1*, *NR2F6*, *FOXC2*) and immunosuppression (*KLF3*, *IRF6*, *TBX21*) in tumor samples as compared with normal pancreata (28–33). In addition, regulon activity corresponding to ONECUT2 increased in epithelial cells from tumor tissue. *ONECUT2* has been implicated in driving neuroendocrine prostate cancer and promoting metastasis in ovarian cancer (34, 35). Tumor-infiltrating T cells expressed higher regulon activity scores for *FOXO* family transcription factors, which mediate induction of renewal capacity in memory T cells and effector function in cytotoxic T cells (36–38). In addition, *NFKB2*, *STAT4*, and *STAT1* regulon activity were increased in T cells from tumor tissue. These transcription factors are critical regulators of innate and adaptive immune responses, T cell effector and memory function, and helper T cell differentiation (39, 40). TAMs exhibited enrichment for *SREBF2* and *PPARG*, regulators of cholesterol and lipid homeostasis, respectively (31, 41). Recent studies have shown that *PPARG* plays a critical role in TAM polarization in the TME and may be an actionable therapeutic target (42, 43).

**Figure 2:**
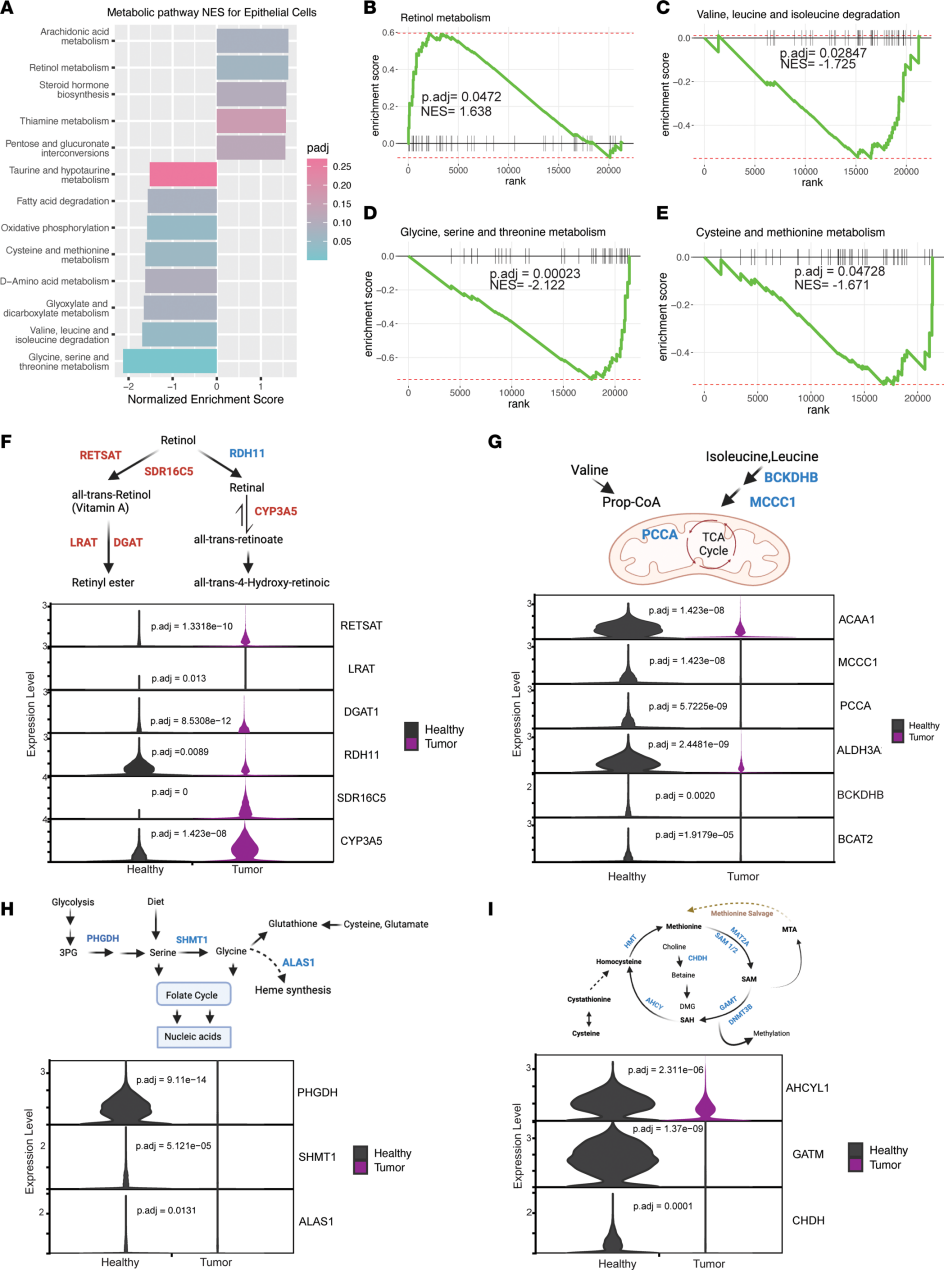
Metabolic coadaptations in pancreatic cancer cells. (**A**) Significantly altered metabolic pathways in epithelial cells derived from pancreatic cancer samples (*n* = 16) compared with healthy pancreas samples (*n* = 6), with corresponding normalized enrichment scores (NES) and adjusted *P* values from GSEA. (**B**–**E**) GSEA enrichment plots of significantly up- or downregulated metabolic pathways in cancer cells with corresponding NES and adjusted *P* values. (**F**) Schematic of retinol metabolism, blue corresponding to differentially decreased genes and red to differentially increased in tumor-derived epithelial cells. Violin plots of selected retinol metabolism genes comparing healthy to tumor, with adjusted *P* values for significantly differentially expressed genes. (**G**) Schematic of valine, leucine, and isoleucine degradation. Violin plots of selected valine, leucine, and isoleucine metabolism genes comparing healthy with tumor, with adjusted *P* values for significantly differentially expressed genes. (**H**) Schematic of glycine, serine, and threonine metabolism. Violin plots of selected glycine, serine, and threonine metabolism genes comparing healthy with tumor, with adjusted *P* values for significantly differentially expressed genes. (**I**) Schematic of cysteine and methionine metabolism. Violin plots of cysteine and methionine metabolism genes comparing healthy with tumor, with adjusted *P* values for significantly differentially expressed genes.

**Figure 3:**
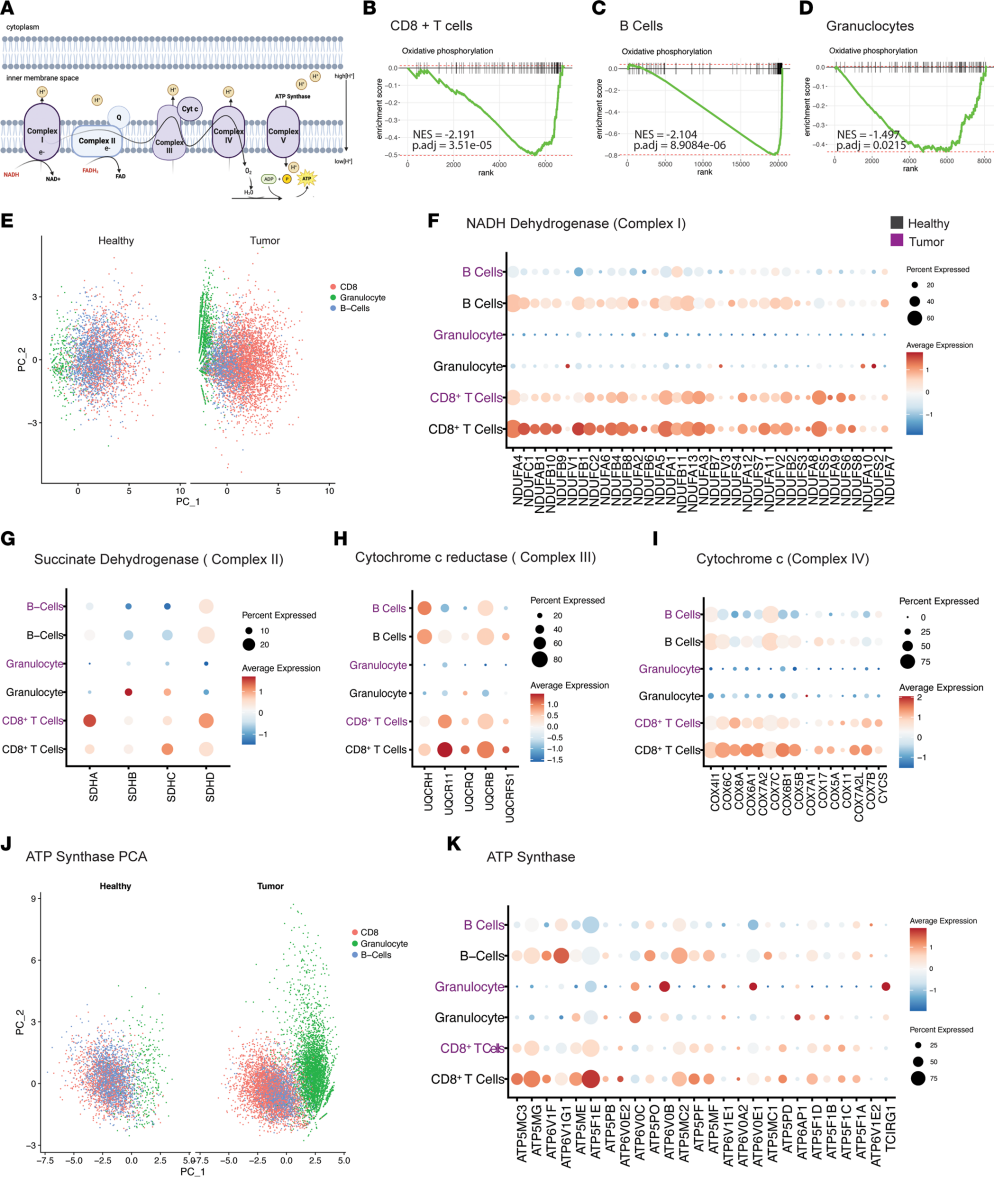
Downregulation of oxidative phosphorylation in immune cells. (**A**) Schematic of electron transport chain (ETC). (**B**–**D**) GSEA enrichment plots demonstrating oxidative phosphorylation is significantly downregulated in CD8^+^ T cells, B cells, and granulocytes derived from PDA samples, compared with healthy human pancreas tissue, with corresponding NES and adjusted *P* values. (**E**) PCA visualization based on the expression of genes driving complex I in B cells, granulocytes, and CD8^+^ T cells in healthy human and PDA samples. (**F**–**I**) Dot plot visualization based on the average expression and percentage of cells expressing genes driving complexes I, II, III, and IV, respectively, in B cells, granulocytes, and CD8^+^ T cells in healthy human (black) and PDA samples (purple). (**J**) PCA visualization based on the expression of genes driving ATP synthase in B cells, granulocytes, and CD8^+^ T cells in healthy human and PDA samples. (**K**) Dot plot visualization of cells expressing ATP synthase–related genes and percentage expressing these genes in immune cells from tumor tissue (purple) and healthy tissue (black).

**Figure 4:**
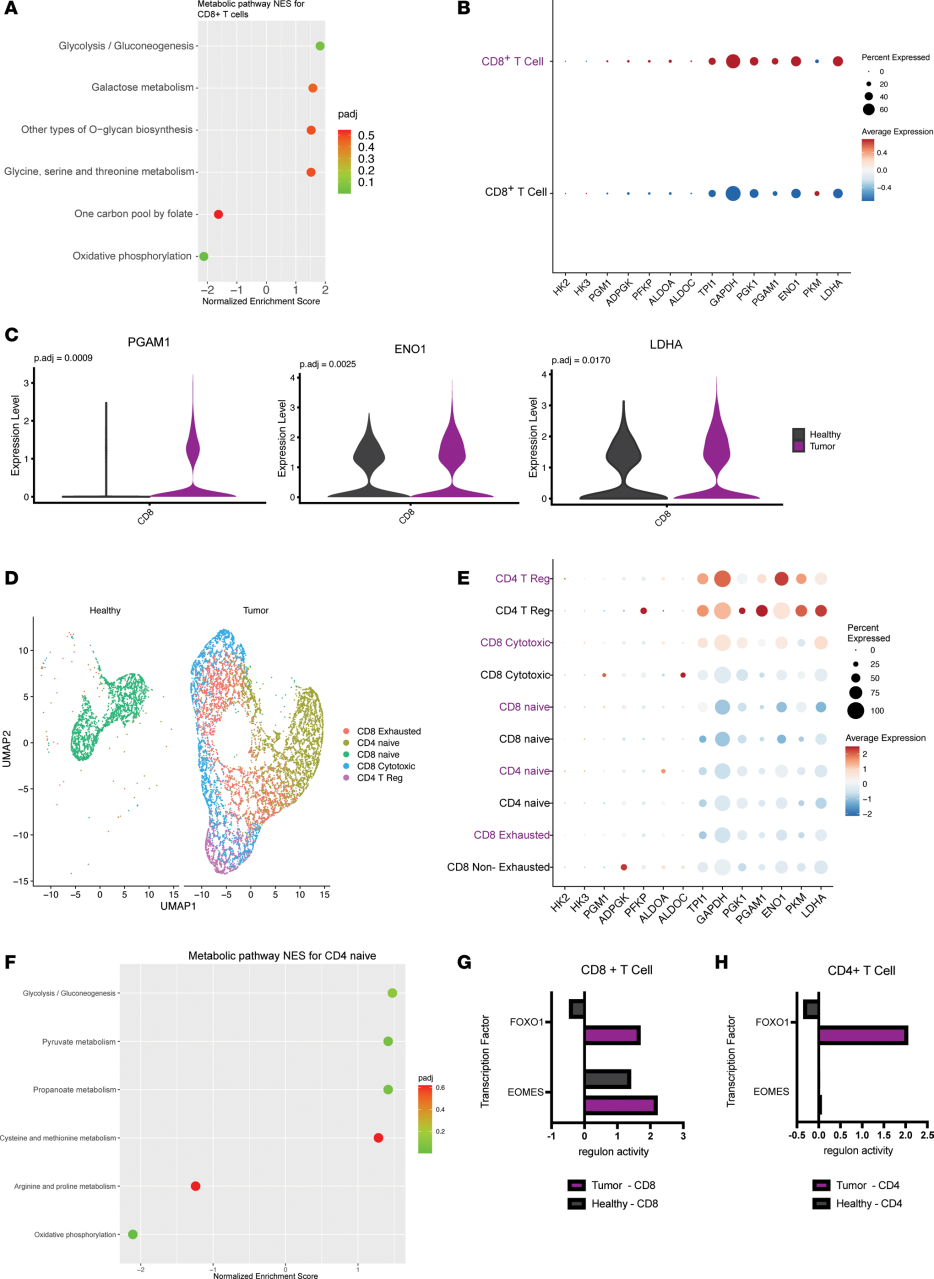
Metabolic rewiring of T cells in the pancreatic cancer microenvironment. (**A**) Significantly altered pathways in CD8^+^ T cells from PDA samples compared with CD8^+^ T cells derived from the healthy tissue. (**B**) Dot plot visualization of the average expression and percentage of cells expressing genes driving glycolysis in CD8^+^ T cells from tumor tissue (purple) and healthy tissue (black). (**C**) Violin plots of the expression of selected differentially expressed glycolysis metabolism genes comparing CD8^+^ T cells from tumor samples with those from healthy samples. (**D**) UMAP visualization of CD4^+^ and CD8^+^ T cell populations in the tumor and healthy tissue. (**E**) Dot plot visualization of the average expression and percentage of cells expressing genes driving glycolysis in CD4^+^ and CD8^+^ T cell populations from tumor tissue (purple) and healthy tissue (black). (**F**) Significantly altered pathways in CD4^+^ naive cells from PDA samples compared with healthy naive CD4^+^ cells. (**G** and **H**) Transcription factor analysis showing regulon activity scores of FOXO1 and EOMES in CD8^+^ T cells in tumor and healthy samples.

Finally, to compare metabolic gene expression programs, we performed GSEA with a curated list of pathways containing all metabolic gene sets available from the Kyoto Encyclopedia of Genes and Genomes (KEGG) database (44). GSEA relies on gene sets to computationally determine statistical significance between 2 states. It is therefore more stringent than DGE analysis and may capture processes not readily apparent with DGE analysis. We focused this analysis on epithelial cells, macrophages, granulocytes, T cells (both CD4^+^ and CD8^+^), and B cells, based on the abundance of cells available for this analysis (Supplemental Figure 1A). As noted above, some cell populations could not be compared as they had limited representation in both healthy and tumor samples. Epithelial cancer cells exhibited higher vitamin A and biosynthetic machinery (Figure 2, B–E), while many immune cell types in the tumor downregulated mitochondrial respiration (Figure 3, B–D), and CD8^+^ T cells in the tumor demonstrated unique metabolic deregulation associated with exhaustion (Figure 4, G and H). These observations are divided on a cell type–specific basis in the sections that follow.

### Pancreatic cancer cells engage vitamin A metabolism and downregulate amino acid catabolism.

We started our investigation into metabolic rewiring with cancer epithelial cells relative to normal epithelial cells (Figure 2A). Cancer cells co-opt a wide array of metabolic adaptations to manage deregulated nutrient and oxygen availability and to disrupt access to antitumor immune cells (45). Similar to prior reports, we observed that pentose conversions were increased in cancer cells, as described previously (6, 46), as were lipid and vitamin metabolism pathways (Figure 2, A and B). Specifically, epithelial cells derived from tumor tissue decreased oxidative phosphorylation and fatty acid oxidation, as well as several amino acid catabolic pathways (Figure 2, A and C–E, and Supplemental Figure 2A) (47, 48).

Next, we examined differential expression of genes that contributed most to the enrichment score, denoted as leading edge genes (Figure 2, F–I). Retinol metabolism was the only significantly increased pathway in tumor-derived epithelial cells compared with healthy epithelial cells (Figure 2B). Within this pathway, we observed that tumor cells differentially increased the expression of genes encoding enzymes related to the production of retinol aldehydes, retinyl esters, and retinoic acid (Figure 2F). Retinoic acid signaling is involved in development, proliferation, and mediation of mechanosensing in the stroma through its interaction with myosin light chain 2 (MLC-2) (49, 50). More specifically, the vitamin A metabolite all-trans retinoic acid has been implicated in reprogramming the PDA stroma via downregulation of *MLC-2*, leading to pancreatic stellate cell quiescence (51). For this reason, targeting vitamin A metabolism has been proposed as a potential therapeutic strategy for stromal reprogramming (52). Our data demonstrate a significant increase in *RETSTAT* expression (Figure 2F), which Bi et al. identified as a crucial mediator of ferroptosis (53). Collectively, our data add to the growing body of research pointing toward retinoic acid signaling as a critical mediator of tumor progression and maintenance, though future functional work is needed to support this possibility.

Amino acid degradation pathways were the most downregulated in epithelial cells from pancreatic tumors, relative to normal epithelial cells (Figure 2, C–E). These included branched chain amino acid (BCAA) metabolism, glycine/serine/threonine metabolism, and cysteine/methionine metabolism. In general, the broad downregulation of amino acid catabolism may suggest increased utilization/prioritization of amino acids for protein biosynthesis. However, each of these pathways serves other functions, and their downregulation may reflect other altered purposes. For example, BCAA catabolism can fuel tricarboxylic acid cycle anaplerosis, a process that also provides nitrogen for other functions (54) (Figure 2G). In either case, our observation is consistent with a recent study that illustrated decreased BCAA degradation in PDA models (23). In a related study, it was also shown that stromal cell reprogramming in PDA can lead to the production and release of BCAAs from stromal cells and their provision to PDA cells (11). These studies and others (54) have focused on the branched chain amino acid transaminase (BCAT); our data showed decreased *BCAT1/2* gene expression in tumor-derived epithelial cells, thereby adding to a growing body of work demonstrating that PDA cells seemingly prioritize BCAAs for purposes other than degradation.

Similarly, glycine/serine/threonine have nonproteinogenic functions. In humans, threonine is not catabolized (55), and the inclusion of this GSEA term was captured based on the functions of glycine and serine. Unlike BCAAs, glycine and serine are nonessential amino acids. They can be obtained through diet or made de novo in most cell types in the body (56). We observed that both serine synthesis (based on *PHGDH* expression) and catabolism (based on *SHMT1* expression) were downregulated in cancerous epithelial cells (Figure 2H), indicating that serine is likely derived from diet or other cell types in pancreatic cancer. Further, glycine and serine are substrates for 1-carbon metabolism, and glycine (which can be derived from serine) is 1 of 3 amino acids in the glutathione tripeptide. Our data also suggest a decreased reliance on serine and glycine for these pathways.

GSEA also revealed a significant decrease in cysteine and methionine metabolism in tumor-derived epithelial cells compared with healthy epithelial cells (Figure 2E). Based on the genes involved, this centered on methionine metabolism and its role in providing 1-carbon units (Figure 2I). The decrease in 1-carbon units from serine/glycine and methionine metabolism indicates either that there is a decrease in histone methylation or that another source of 1-carbon units stands in for these amino acids. Collectively, these observations suggest decreased amino acid catabolism in the cancer cells may support protein biosynthesis. It is important to note that future studies with additional samples might increase statistical power, leading to the identification of additional metabolic pathways altered in cancer cells.

### Differential repression of oxidative phosphorylation machinery across immune populations in pancreatic tumors.

Next, we sought to determine how innate and adaptive immunity is metabolically shaped by the TME. We observed that multiple immune compartments in pancreatic cancer samples decreased their oxidative phosphorylation signature (Figure 3A) compared with healthy human pancreas tissue. Most prominent among these were CD8^+^ T cells, B cells, and granulocytes (Figure 3, B–D). Next, we assessed the leading edge genes per cell type and per ETC complex (Figure 3, E–K). As shown in Figure 3E, we performed PCA based on gene expression of the 44 subunits in complex I. Remarkably, we found that expression levels of genes encoding subunits of complex I distinguished immune compartments from one another in the tumor condition, without lineage markers (Figure 3E). In other words, the metabolic signature of each immune cell population in the tumor is as distinct as the canonical cell surface markers used to define immune cells.

Next, we segregated immune compartments in the tumor condition based on average expression and percentage of cells expressing genes that encode the subunits of complexes (Figure 3, F–I).

Complex I–encoding gene expression components were downregulated to varying degrees across all immune compartments, compared with their counterparts in healthy pancreata (Figure 3F). Most notably, B cells in the tumor decreased expression of multiple genes encoding proteins that drive complex I. Meanwhile, complex I genes were not highly expressed in granulocytes, nor did they differ significantly based on sample condition. Last, tumor CD8^+^ T cells exhibited decreased expression of a few genes in complex I. In complex II, the second entry into the ETC, tumor-associated granulocytes decreased expression of succinate dehydrogenase isoforms *SDHC* and *SDHB* (Figure 3G). To our knowledge, dysregulation of complex II in tumor-associated granulocytes has not been shown in pancreatic cancer. In contrast, B cells did not display a shift in expression of complex II genes in the tumor compared with healthy tissue (Figure 3G). CD8^+^ T cells showed a slight increase in the expression of *SDHA* and *SDHD* in tumors (Figure 3G).

Differences in expression based on cell type in the tumor compared with healthy pancreas were also seen in complexes III and IV (Figure 3, H and I). Notably, B cells had the greatest decrease in expression of genes encoding complexes III and IV. Segregation based on expression and percentage of cells expressing complex IV and III genes, respectively, did not display a pattern in the tumor condition, unlike our findings in complex I (Supplemental Figure 3, A and B). However, expression of leading edge genes related to ATP synthase, i.e., complex V, led to segregation of the immune compartments in the tumor condition (Figure 3J). As previous trends demonstrated, the degree of downregulation and the leading edge genes that decreased in expression were cell type dependent for complex V (Figure 3K). Each immune compartment followed a different pattern of expression in tumor compared with healthy tissue. Importantly, we assessed read coverage of glycolytic genes across immune compartments and found adequate coverage, and yet tumor-associated B cells and granulocytes did not significantly alter glycolysis relative to the normal pancreas (Supplemental Figure 3, C–E). In contrast, there was a marked upregulation of glycolytic gene expression in CD8^+^ T cells, as is discussed below. The increase in glycolytic gene expression beginning at *GAPDH* is reflective only of CD8^+^ T cells, not granulocytes and B cells (Supplemental Figure 3, F and G). Collectively, this may suggest that tumor-associated B cells and granulocytes do not shift toward glycolytic dependence in the same manner as CD8^+^ T cells.

### Metabolic rewiring of T cells.

Intrigued by the marked decrease in ETC complex expression, we next assessed more globally the metabolic differences between tumor-derived CD8^+^ T cells and CD8^+^ T cells from the healthy pancreas by performing GSEA (Figure 4A). In agreement with previous studies of T cells in solid tumors, tumor-derived CD8^+^ T cells showed significant upregulation of glycolysis (57, 58) (Figure 4A). However, unlike classical descriptions of CD8^+^ T cell differentiation and expansion, the increase in glycolytic gene expression was accompanied by a decrease in oxidative phosphorylation (Figure 3B and Figure 4A). We put forth that this dichotomous activation of bioenergetic pathways is the likely result of low oxygen availability in the pancreatic TME, based on the observed hypoxia signature in CD8^+^ T cells (Supplemental Figure 4, A and B). Further, analysis of expression of individual glycolytic enzymes demonstrated an increase in expression of genes downstream of *GAPDH* (Figure 4, B and C). We hypothesize that this occurs to assist in clearance of reductive stress to facilitate continued glycolysis in low-oxygen conditions. When CD8^+^ T cells were subset into exhausted, cytotoxic, and naive populations, no significant metabolic alterations were seen between conditions (Supplemental Figure 4, D–F). It is important to note that since sample sizes decreased when subsetting CD8^+^ T cell populations, these comparisons are less robust. At the population level, CD4^+^ T cells did not exhibit significant differences in gene expression between the tumor condition and healthy pancreas. Thus, we subclustered the CD4^+^ T populations to gain a more granular view (Figure 4D). We used previously published markers to delineate the various subtypes of T cells (Supplemental Figure 4C). Subclustering CD8^+^ and CD4^+^ T cells revealed additional insights (Figure 4E); for example, naive CD4^+^ T cells exhibited metabolic changes in the TME that largely mirrored those in bulk CD8^+^ T cell populations (Figure 4F).

To interrogate master regulators of transcriptional programs in T cells, transcription factor analysis was performed using the SCENIC package in R. The activity of transcription factor target genes corresponds to regulon activity, where a higher score indicates a higher inferred activity level of target genes. The strongest scoring transcription factor for this analysis was *FOXO1*. We observed that CD8^+^ T cells and CD4^+^ T cells derived from tumors showed increased *FOXO1* regulon activity scores in comparison with CD4^+^ and CD8^+^ cells derived from healthy pancreas tissue (Figure 4, G and H). *FOXO1* is critical for the activation of memory T cells capable of reexpansion in response to antigen presentation (36, 37, 59). In this capacity *FOXO1* mediates glycosylation (38), whose pathways we observed to be highly differentially regulated in the GSEA (Figure 4A).

Second to *FOXO1* was *EOMES*, a transcription factor involved in the regulation of memory and regulatory T cell function and homeostasis. Increased expression of *EOMES* has been observed in a terminally exhausted subset of infiltrating CD8^+^ T cells (60). Interestingly, *TBX21* regulon activity was increased in tumor-derived T cells (Supplemental Data 3). TBX21 is a critical transcription factor in chronic infection and has been shown to promote a terminally exhausted phenotype in T cells (33, 60). Collectively, our data suggest that a population of tumor-associated T cells may be on a trajectory toward progenitor or terminal exhaustion, unable to execute tumor clearance. This is consistent with previous observations, including by our group, that T cells in pancreatic cancer are dysfunctional (22, 61, 62). Overall, the metabolic profiles of T cell subsets derived from PDA samples suggest a dysfunctional phenotype, marked by hypoxia, decreased oxidative phosphorylation, and a compensatory increase in glycolysis. These results provide a more detailed understanding of how the pancreatic TME deregulates CD8^+^ T cell metabolism and thus function, as well as insight into how T cells compensate.

### Metabolic alterations in TAMs.

**Figure 5:**
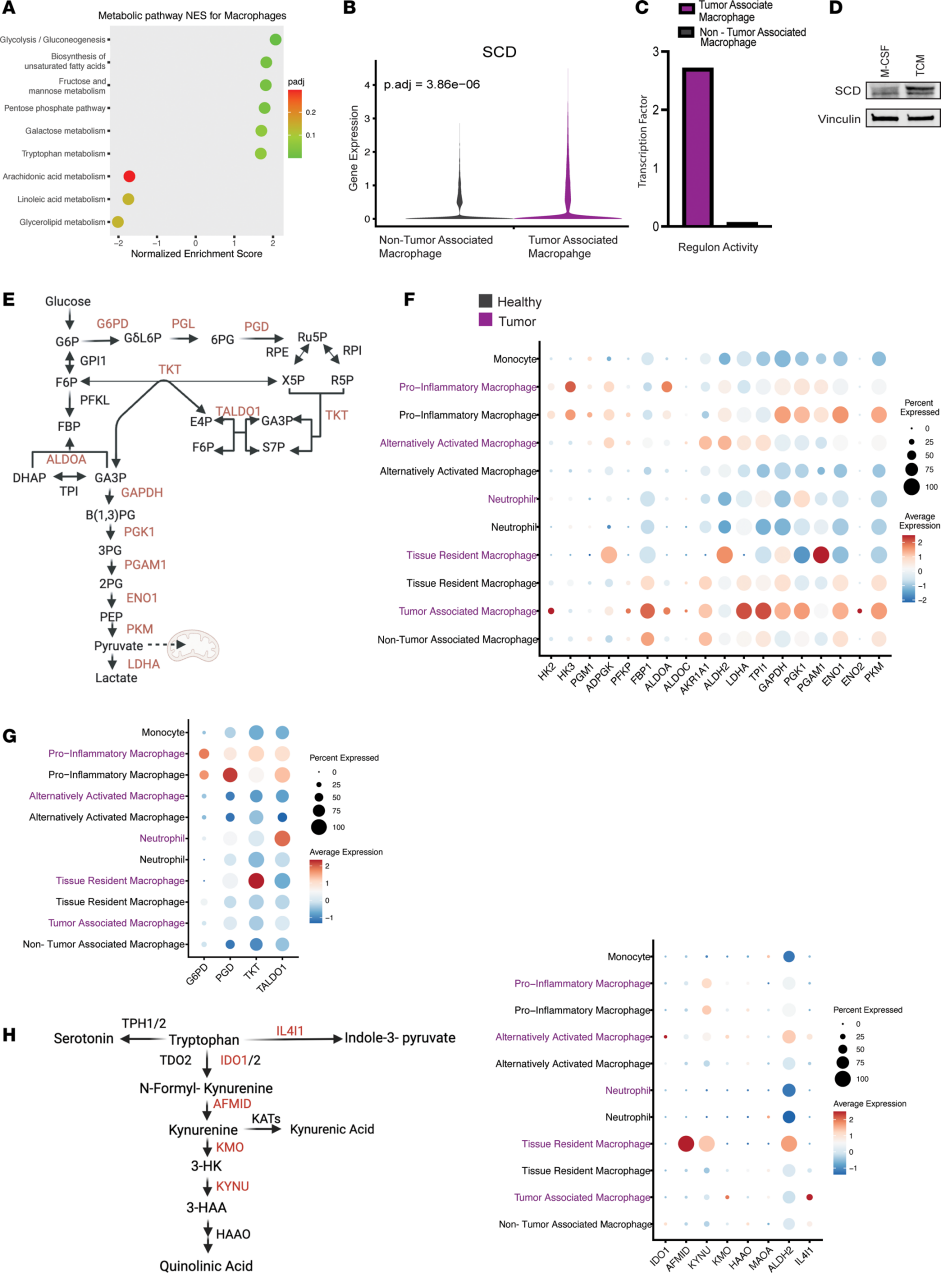
Metabolic alterations in TAMs. (**A**) Significantly altered metabolic pathways in macrophages derived from pancreatic cancer samples compared with healthy pancreas samples, with corresponding NES and adjusted *P* values. (**B**) Violin plot of the expression of stearoyl-CoA desaturase (SCD) in macrophages in tumor and healthy samples, showing differential expression. (**C**) Transcription factor analysis showing regulon activity of PPARG in macrophages in tumor and healthy samples. (**D**) Western blot, where protein expression of SCD is higher in murine bone marrow–derived monocytes treated with TCM compared with control condition with M-CSF. (**E**) Glucose and PPP schematic. (**F**) Dot plot visualization of genes driving glycolysis displaying average expression and percentage expressed in macrophages in tumor (purple) and healthy pancreas tissue (black). (**G**) Dot plot visualization of genes driving PPP that do not overlap with glycolysis, displaying average expression and percentage expressed macrophages in tumor (purple) and healthy pancreas tissue (black). (**H**) Tryptophan metabolism schematic, with dot plot visualization of genes driving tryptophan metabolism, displaying average expression and percentage expressed in macrophages in tumor (purple) and healthy pancreas tissue (black).

Macrophages play important roles in healthy tissues, and many metabolic crosstalk features have been documented in the TME. In PDA, TAMs dictate milieu composition, immunosuppressive programs, and efficacy of therapeutic agents (14, 20, 63, 64). To begin our interrogation into macrophages, we first investigated significantly increased or decreased metabolic pathways in TAMs compared with macrophages in the healthy pancreas (Figure 5A). In TAMs, we observed that glycolysis, the pentose phosphate pathway (PPP), unsaturated fatty acid synthesis, and fructose and mannose metabolism were significantly increased. This is consistent with previous studies pointing to altered carbohydrate, lipid, and amino acid metabolism in TAMs (20, 63, 65).

We then observed that TAMs upregulated the expression of several enzymes that drive unsaturated fatty acid synthesis, including *ACOT2/4/7*, *HACD4*, and stearoyl-CoA desaturase (*SCD*) (Figure 5B and Supplemental Figure 5A). In addition, we found *PPARG* regulon activity to be increased in TAMs compared with healthy macrophages based on *PPARG* regulon activity (Figure 5C). As upregulation of unsaturated fatty acid synthesis enzymes in pancreatic TAMs has not been previously reported, we employed a murine model of pancreatic TAM polarization to assess whether *Scd1*, a key enzyme in this pathway, was expressed at the protein level. In brief, we isolated bone marrow–derived monocytes and polarized them with tumor-conditioned media (TCM); this approach activates expression of hallmark genes of TAMs (15). Polarization with TCM promoted increased *Scd1* expression in TAMs at the protein level (Figure 5D). These results indicate that TAMs upregulate components of the unsaturated fatty acid synthesis pathway in response to cancer cell signals. TAMs can display considerable cellular plasticity, dependent on exogenous signaling factors in their environment. To explore this, we next subclustered macrophage populations for higher resolution analysis using markers reflecting current classification paradigms (63, 66) (Supplemental Figure 5, B and C). To better visualize the changes between macrophage subsets in tumor compared with normal tissue, we assessed the following pathways: glycolysis, PPP, and tryptophan catabolism, based on the percentage of cells expressing genes related to each significantly altered pathway, alongside average expression (Figure 5, E–H).

TAMs exhibited the greatest increase in glycolysis in tumor samples compared with healthy counterparts (64) (Figure 5F). Monocyte-derived cells also trended toward an increase in glycolysis. Consistent with precedent, pro-inflammatory macrophages derived from both tumor and healthy pancreas expressed genes driving the PPP to a greater degree than the other macrophage subtypes (Figure 5G). Next, we looked at tryptophan metabolism in macrophage subtypes, as it was borderline significant in our analysis and is a well-known metabolic pathway in macrophages (65, 67, 68). The shift toward tryptophan metabolism in tissue-resident macrophages derived from tumor compared with healthy pancreas exhibited several notable features (Figure 5H and Supplemental Figure 5E). Indeed, we observed a combination of increased tryptophan catabolism, based on IL4I1 expression, and a marked increase in arylformamidase (*AFMID*), which yields kynurenine, a well-known metabolic suppressor of T cell function and activation (12, 64, 69). Last, we performed GSEA on each subpopulation of macrophages and found significantly increased glycolysis and oxidative phosphorylation metabolic signatures in alternatively activated macrophages (Supplemental Figure 5E). In contrast, the remaining macrophage subsets in pancreatic cancer samples did not have significantly altered metabolic programs compared to healthy pancreas (Supplemental Figure 5, F–H), demonstrating that TAMs engage in heterogeneous metabolic activities in the TME that are not restricted to either pro- or antitumor programs.

### Cellular crosstalk between epithelial cells and TAMs.

**Figure 6:**
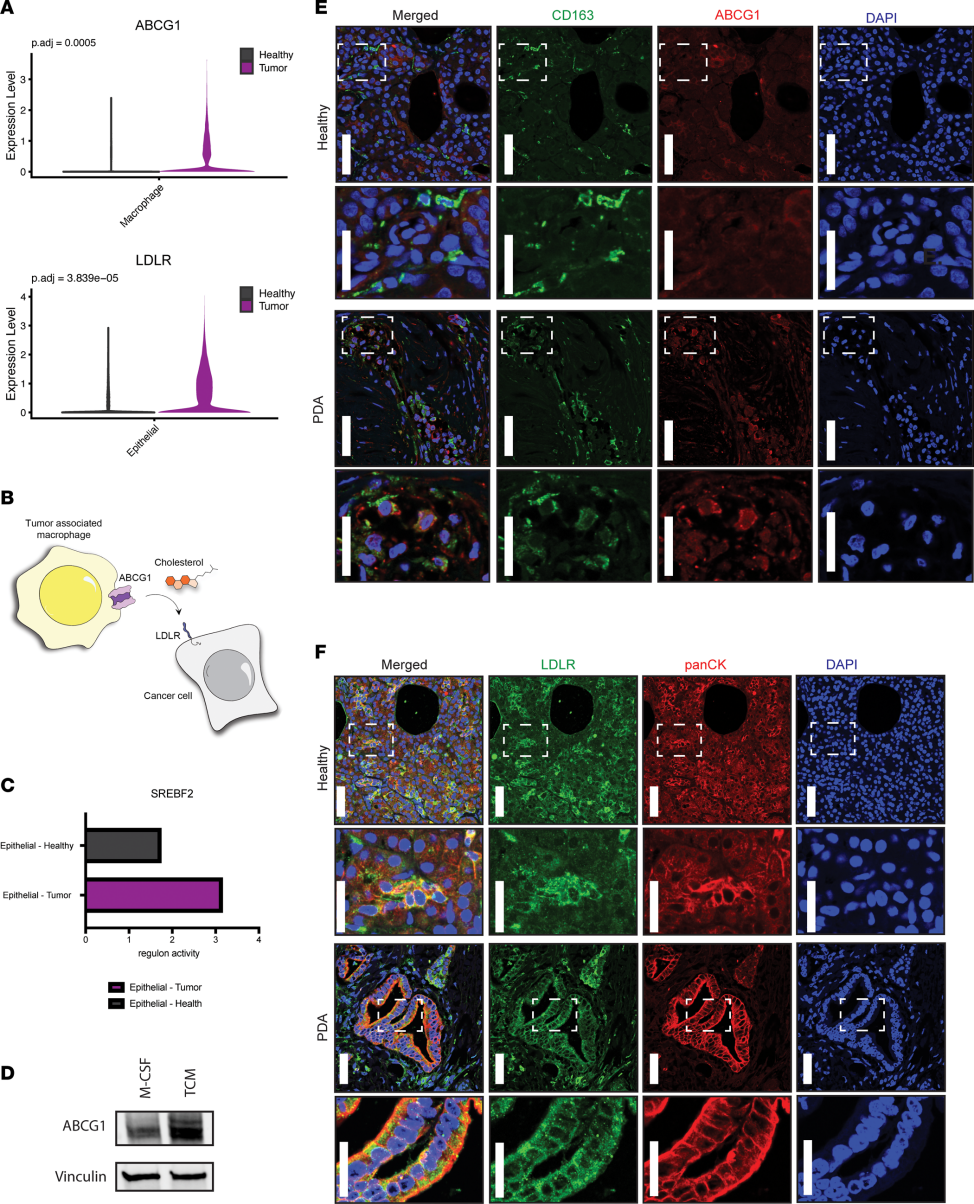
Metabolic cellular crosstalk between epithelial cells and TAMs. (**A**) Violin plots showing ABCG1 is significantly upregulated in TAMs, and LDLR is significantly upregulated in tumor-derived epithelial cells. (**B**) Schematic of TAMs increasing ABCG1 (cholesterol exporter) expression. Cancer cells increase expression of a corresponding lipid/cholesterol receptor LDLR. (**C**) Transcription factor analysis showing SREBF2 regulon activity score is increased in tumor-derived epithelial cells. (**D**) Western blot, where protein expression of ABCG1 is 1.7 times higher in murine bone marrow–derived monocytes treated with TCM compared with control condition with M-CSF. (**E**) Immunofluorescence of CD163 (green), ABCG1 (red), and DAPI (blue) in healthy human pancreas and PDA. (**F**) Immunofluorescence of LDLR (green), panCK (red), and DAPI (blue) in healthy human pancreas and PDA. Immunofluorescence from **E** and **F** is representative of 3 healthy individuals and 3 individuals with PDA, with staining performed twice per sample. For the low-magnification images, the scale bar is 50 μm, and for the zoomed insets, the scale bar is 25 μm.

Pancreatic tumors have limited functional vasculature. Thus, cells in the tumor have varied and constrained access to serum-derived nutrients and oxygen. Numerous reports have detailed compensatory metabolic cross-feeding pathways where cancer cells capture nutrients from other noncancer cell types present in the TME to sustain cellular proliferation and tumor growth (12). We investigated whether we could identify putative cross-feeding pathways from our datasets based on differential pathway activity or importer/exporter expression between cellular compartments that could result in a symbiotic relationship when considered together. Application of this approach led us to identify increased expression of the cholesterol exporter *ABCG1* in TAMs relative to macrophages derived from healthy pancreatic tissue (Figure 6A). This observation suggests that TAMs release more cholesterol. Next, we found that *LDLR* was differentially increased in cancer cells (Figure 6A), suggesting that cancer cells may selectively import cholesterol (Figure 6B).

The transcription factor *SREBP2* activates genes involved in cholesterol synthesis and efflux and expression of the LDLR (31). We assessed the regulon activity score associated with *SREBF2*, the transcript encoding *SREBP2*, and found it to be higher in epithelial cells derived from PDA compared with healthy tissue (Figure 6C). To assess if cancer cell–derived factors play a functional role in TAM *Abcg1* expression, we utilized the in vitro mouse model of pancreatic TAM polarization described above. Treatment of unpolarized macrophages with cancer cell–conditioned media boosted *Abcg1* expression in TAMs by Western blot, relative to that in unpolarized macrophages (Figure 6D). Next, we set out to corroborate these findings at the protein level in human samples. We stained normal pancreas and pancreatic tumor tissue for macrophage-specific (CD163^+^) expression of ABCG1. Indeed, ABCG1 expression was higher in TAMs compared with non-TAMs present in healthy human pancreatic tissue (Figure 6E). In parallel, human pancreatic cancer tissue and healthy human pancreata were probed for LDLR expression in epithelial cells (pan-cytokeratin positive, panCK^+^). LDLR expression was elevated in human pancreatic cancer tissue compared with healthy human pancreatic tissue (Figure 6F). Collectively, these data suggest that pancreatic TAMs may provide cholesterol to cancer cells, as has been previously described in prostate and breast cancer (70, 71).

## Discussion

Cancer and immune cells acquire a wide array of metabolic adaptations, including autonomous and symbiotic adaptations, to circumvent the nutrient-deregulated conditions in the TME, sustain increased bioenergetic demands, and engage in competition for scarce fuel sources (13, 45, 69, 72). Since cancer cells participate in immune-metabolic crosstalk in the TME, oncogenic signaling can both directly and indirectly affect immune cells. This leads to metabolic alterations also engendered in several immune compartments (43, 69, 73). Previous studies have shown immune cell dysfunction in PDA attributed to exhausted T cells unable to execute effector functions, regulatory T cell activity hindered by interactions with TAMs excreting kynurenine, and other signaling pathways co-opted by cancer cells (30, 55, 74, 75). Metabolism directly informs the functional phenotypes of every cell present in the microenvironment.

The advent of next-generation single-cell sequencing technology has enabled the mapping of gene expression, metabolic pathways, and potential cellular interactions in the TME at high resolution (76, 77). scRNA-Seq has been employed to query metabolic heterogeneity in TAMs from other tumor models, where a correlation between metabolic phenotype and function in murine models was observed (78). Nevertheless, access to patient tumor tissue for investigation using single-cell techniques remains difficult, especially from organs not routinely sampled for medical procedures (79, 80). Adjacent normal pancreatic tissue, often used as control, is not “normal,” as it is affected by inflammation and desmoplasia in the pancreas. The availability of the “true normal” allowed us to map out gene expression changes linked to malignancy and specifically query metabolic alterations across all cellular compartments, thereby, building a metabolic atlas from healthy and pancreatic tumor tissue.

Here, we characterize metabolic rewiring of malignant, nonmalignant, and immune cells in the healthy human pancreas compared with human PDA cancer samples. Our findings serve as a resource atlas for understanding the various pathways co-opted by pancreatic cancer cells to maintain survival, while detailing immune cell rewiring and crosstalk in response to oncogenic signaling. Many metabolic studies have leveraged in vitro systems, which enable manipulation of media and metabolite levels and can include select stromal and immune cells (5, 8, 9, 20, 65, 81–86). These conditions do not fully recapitulate physiological circumstances in which the TME milieu contributes to metabolic dysregulation and competition for bioenergetic substrates, nor do they account for the complexities of immunosuppression.

Accordingly, this resource atlas could also be of value to compare and contrast metabolic rewiring of the TME in other cancers and immunometabolism in other healthy and diseased states, for but not only for researchers interested in the truly healthy pancreas. Our work suggests that mitochondrial respiration is downregulated across multiple immune compartments: CD8^+^ T cells, B cells, and granulocytes. This significant decrease in oxidative phosphorylation dependency may be attributed to hypoxic regions in the tumor, a hallmark feature of PDA (85, 86). Limited oxygen availability may pressure immune cells to downregulate ETC dependency (87, 88). Interestingly, the manner in which immune cells shift dependency is not homogenous — rather, it is ETC complex and cell type specific. Remarkably, our work shows complex I subunit expression is reduced in B cells, complex II is decreased in granulocytes, and ETC complex expression is more uniformly decreased in CD8^+^ T cells. Recent work demonstrated that the oxidative flow of electrons through the ETC plays a critical role in cancer cell immune evasion in melanoma. In melanoma, loss of complex II gene expression leads to increased succinate levels; in turn, increased succinate levels drive expression of genes related to antigen presentation and processing through an epigenetic mechanism (89). Or stated reciprocally, enhanced complex I activity promotes antitumor immune cell recognition. We believe our data reveal for the first time that complex I subunit expression is decreased in B cells in pancreatic cancer. This sets precedence for the investigation of electron flow manipulation employed by immune cells in PDA and how this may contribute to immunosuppression. In addition, nutrient scarcity, as well as the secreted products of altered metabolism in cancer cells, shifts phenotypes of innate immune cells, which are supported by metabolic processes (57). Overall, our data show a comprehensive analysis across immune compartments that points to mitochondrial immune dysfunction in PDA. Intriguingly, the mechanisms of oxidative phosphorylation downregulation are cell type specific. Future work will be needed to elucidate the causes and functional consequences of this observation.

TAMs are a metabolically heterogeneous group of cells that engage in cellular exchange of nutrients and metabolites with cancer cells present in the TME. Our work agrees with previous findings and sheds light on metabolic changes and interactions. We found that TAMs increased expression of the unsaturated fatty acid synthesis protein SCD1 in vitro and that this was regulated by factors secreted from tumor cells. Thus, we became interested in investigating metabolic exchanges advantageous to cancer cells. For example, a recent study illustrated that TAMs transfer cholesterol to cancer cells, conferring therapeutic resistance in castration-resistant prostate cancer by modulating cholesterol/androgen signaling (70). This phenomenon was demonstrated in a breast cancer model, driven by *IL4*-mediated signaling (71). A similar crosstalk between TAMs and transformed epithelium takes place in mutant EGFR–driven lung adenocarcinoma, leading to metabolic rewiring and pro-inflammatory response in tumor-associated alveolar macrophages (90).

These findings, together with altered lipid metabolism in TAMs in our data, prompted us to investigate the reciprocal relationship between the increased expression of the ABCG1 cholesterol exporter on TAMs and the cognate lipid/cholesterol receptor on pancreatic cancer cells. Through co-immunofluorescence staining in healthy human tissue and pancreatic cancer samples, we found increased ABCG1 expression in macrophages and increased LDLR in pancreatic cancer samples. To our knowledge, this is the first report suggesting pancreatic cancer cells engage in cholesterol exchange with TAMs. How this crosstalk may mediate an immunosuppressive signaling axis in pancreatic cancer remains to be further investigated.

It is important to acknowledge limitations of the current study. The nature of sample acquisition, in particular for biopsy samples but also when larger tissue pieces are acquired, is that not all areas of a specimen can be analyzed; pancreatic cancer is notoriously heterogeneous, specifically where the desmoplastic stroma is concerned (91). Future studies utilizing emerging technologies such as spatial metabolomics might elucidate metabolic “neighborhoods” in individual samples. In addition, future work will be needed to dissect the functional consequences of metabolic reprogramming in each cellular compartment.

## Methods

### Sex as a biological variable

Pancreatic cancer has similar incidence in males and females. scRNA-Seq data from both pancreatic cancer samples and normal pancreata contain similar numbers of samples from male and female individuals.

### Donor sample procurement and tissue processing

Donor pancreata were collected at the Gift of Life Michigan Donor Care Center and preserved as previously published in Carpenter et al., 2023 (21). Briefly, portions of the dissected pancreas (head, body, and tail) were each placed into DMEM with 1% BSA/10 μmol/L Y27632 or 10% formalin for single-cell sequencing or paraffin embedding, respectively. Further processing was done to prepare the samples for single-cell processing: mince tissue into 1 mm^3^ pieces, digest with 1 mg/mL collagenase P (Roche) for 20 to 30 minutes at 37°C with gentle agitation, rinse 3 times with DMEM/1% BSA/10 μmol/L Y27632, and then filter through a 40 μm mesh (Falcon, Thermo Fisher Scientific). Resulting cells were submitted to the University of Michigan Advanced Genomics Core for single-cell sequencing using the 10x Genomics Platform.

### PDA patient samples

Resected PDA from patients seen at the University of Michigan Health System from 2021 to 2022 were included in this study, as described previously in Steele et al., 2020 (22). Tissues were fixed in 10% neutral buffered formalin and paraffin embedded using standard protocols before sectioning and staining. All hematoxylin and eosin–stained slides were reviewed, diagnoses confirmed, and corresponding areas carefully selected and marked.

### scRNA-Seq

The samples were run on the 10x Genomics platform, and subsequent analysis was previously described and published by Carpenter et al., 2023 (21). To subset the T cell population, as well as the myeloid population for higher resolution of cell types, markers from previously published studies were utilized to annotate subpopulations.

### Pseudobulk RNA DGE

As previously published in Carpenter et al., 2023, counts were aggregated from all the different samples or for a subset of cells (21). Counts were corrected by removing background contamination signal and transformed to integers. To aggregate to the sample level, the mean function was utilized. For normalization and DGE analysis of the samples, the DESeq2 package from R was used. Dimensionality reduction was employed. Better visualization of differences between groups was performed using the PCAtools package from R.

### Metabolic pathways and GSEA

Selected metabolic pathways were retrieved from the KEGG database: https://www.genome.jp/kegg/pathway.html#metabolism

The list of pathways was downloaded using a bash script, and the pathway to gene mapping was downloaded from the KEGG database at https://rest.kegg.jp/link/pathway/hsa GSEA was performed using the GSEABase R package. For GSEA, the fgsea package in R was used together with the metabolic gene sets downloaded from KEGG. This analysis was performed to identify significantly enriched metabolic pathways.

### Transcription factor inference analysis

Transcription factor inference analysis was performed using SCENIC (v1.3.1) per cell type with raw count matrices corresponding to tumor and healthy tissue, respectively. The regulons and transcription factor activity (AUC) per cell was calculated with the pySCENIC program (v 0.12.1) with motif collection version mc9nr.

### Cell culture (bone marrow–derived monocyte isolation + TCM)

#### Conditioned media.

To make fibroblast-conditioned media or TCM, L929 fibroblasts (American Tissue Culture Collection) or 7940b tumor cells (a gift from Gregory Beatty, University of Pennsylvania, Philadelphia, Pennsylvania, USA) were cultured in DMEM (Thermo Fisher Scientific, catalog 11965-092) with 10% FBS, and media were collected after 48 hours of growth. Media were centrifuged at 200*g* for 5 minutes and passed through a 0.22 μm filter to remove cells.

#### Bone marrow–derived monocyte isolation/polarization.

To make macrophage growth media, fibroblast-conditioned media were added at 30% volume to DMEM. Bone marrow cells were then harvested from WT C57BL/6 mouse (Jackson Laboratories) femurs and tibias and cultured in macrophage growth media for 7 days. Fresh media were added on days 2 and 5, and cells were reseeded in 6-well plates on day 6 to prepare for polarization.

On day 7, media were replaced with polarization media, which consisted of DMEM with M-CSF (10 μg/mL, PeproTech), LPS (10 μg/mL, Thermo Fisher Scientific), IL-4 (PeproTech), or 50% TCM. Polarized macrophages were harvested after 48 hours for analysis by Western blot.

### Western blot analysis

Protein was isolated from cells with RIPA Lysis and Extraction Buffer (Thermo Fisher Scientific). Isolated protein was quantified and normalized via Pierce BCA assay (Thermo Fisher Scientific). A total of 60 μg protein was run on 4%–15% SDS-PAGE gels and transferred onto nitrocellulose membranes (Invitrogen, Thermo Fisher Scientific). Membrane was blocked with 5% BSA (Thermo Fisher Scientific) in Tris-buffered saline–Tween buffer. The membranes were probed with the following antibodies: anti-ABCG1 (rabbit) Proteintech, catalog 13578-1-AP; anti-SCD1 (mouse), Invitrogen, Thermo Fisher Scientific, clone CD.E10; and anti-Vinculin (rabbit) Cell Signaling Technology, catalog 13901S.

### Immunofluorescence

FFPE normal pancreas and pancreatic cancer tissue sections were mounted onto glass slides, deparaffinized, dehydrated in graded ethanol, and rinsed in deionized water. Slides were quenched with hydrogen peroxide solution for 15 minutes and washed with PBS. Antigen retrieval was performed with 10 mM sodium citrate buffer at pH 6.0 with 0.05% Tween 20 for 30 minutes at 96°C. Slides were cooled to room temperature and washed 3 times with PBS.

For co-immunofluorescence with primary antibodies made in the same animal (ABCG1 and F4/80) the Tyramide SuperBoost Kit with Alexa Fluor Tyramide (Invitrogen, Thermo Fisher Scientific, catalog number B40922) was used per manufacturer’s protocol. Briefly, tissues were blocked with kit blocking buffer (Component A) for 1 hour at room temperature, and the first primary antibody was added and incubated overnight at 4°C in PBS with 2.5% BSA and 0.2% Triton X-100. Slides were rinsed with PBS, and kit HRP-conjugated streptavidin (Component B) was added for 1 hour at room temperature. Tyramide working solution was added for 10 minutes followed by Reaction Stop Reagent working solution for 5 minutes. After rinsing in PBS, slides were boiled in 10 mM sodium citrate buffer at pH 6.0 with 0.05% Tween 20 for 20 minutes. Slides were cooled and blocked in kit blocking buffer (Component A) for 1 hour at room temperature. Additional primary antibodies were incubated overnight at 4°C in PBS with 2.5% BSA and 0.2% Triton X-100. Slides were rinsed with PBS and secondary antibodies were added for 1 hour at room temperature. DAPI was added to slides for 10 minutes at room temperature. Slides were rinsed in PBS and mounted in ProLong Gold Antifade Mountant (Invitrogen, Thermo Fisher Scientific).

For co-immunofluorescence with primary antibodies made in different animals, slides were blocked for 1 hour at room temperature in PBS with 2.5% BSA and 0.2% Triton X-100. Primary antibodies were incubated overnight at 4°C. The next day, slides were rinsed and incubated with secondary antibodies for 1 hour at room temperature. DAPI was added for 10 minutes at room temperature. Slides were rinsed in PBS and mounted in ProLong Gold Antifade Mountant.

The following antibodies were used: anti-ABCG1 (rabbit), Proteintech, catalog 13578-1-AP; anti-CD163 (mouse), Novocastra, NCL-L-CD163; anti-LDLR, Invitrogen, Thermo Fisher Scientific, catalog MA5-32075; and anti-PanCytokeratin (mouse), BioLegend catalog 628602. The following secondary antibodies were also used: Alexa Fluor 488 (goat anti-rabbit), Thermo Fisher Scientific, catalog 327731; Alexa Fluor 594 (goat anti-rabbit), Invitrogen, Thermo Fisher Scientific, catalog A-11012; and Alexa Fluor 555 (goat anti-mouse), Invitrogen, Thermo Fisher Scientific, catalog A32727.

High-magnification images were obtained on a Leica Stellaris confocal microscope at the University of Michigan Biomedical Research Core Facilities Microscopy Core.

### Statistics

The DESeq2 R package was used for DGE analysis, and it uses a 2-tailed Wald test to compute a *P* value for each gene that is the probability to get a Wald test statistic as extreme or more extreme as the one observed if the gene is not differentially expressed. Correction for multiple comparisons using the Benjamini and Hochberg method is employed by default in the DESeq2 package to control for the false discovery rate. The *P* values are reported. The GSEA method as implemented in the fgsea R package was used for GSEA, and it computes a running sum statistic that estimates the association of the gene set with the phenotype under study. A *P* value associated with this statistic is also computed using a method described as a multilevel split Monte Carlo simulation (https://rdrr.io/bioc/fgsea/man/fgseaMultilevel.html). The *P* values are then corrected using the Benjamini and Hochberg method, which is the default one in the gsea package. The Seurat R package is used for differential expression at the single-cell level to find markers, using a Wilcoxon rank sum test for *P* value calculation and the Bonferroni correction for multiple comparisons. *P* < 0.05 was considered statistically significant.

### Study approval

The research project and protocol for donor sample acquisition were approved by the Gift of Life research review group (Ann Arbor, Michigan, USA). The protocol was previously described and approved by the University of Michigan Institutional Review Board (HUM00025339). The collection of patient-derived tissues for histological analyses was approved by the Institutional Review Board at the University of Michigan (HUM00098128). Mouse studies were approved by the Institutional Animal Care and Use Committee at the University of Michigan (PRO00011612).

### Data availability

Human scRNA-Seq data were previously published in Steele et al., 2020 (22) (NCBI dbGaP database accession phs002071.v1.p1). Human scRNA-Seq data were previously published in Carpenter et al., 2023 (21). Raw scRNA-Seq data from donor pancreata are available at the NCBI dbGaP database under the accession phs003229. Feature matrices of scRNA-Seq data are available at accession number GSE229413. Supporting data for Figures 5 and 6 can be found in the Supporting Data Values file.

## Author contributions

MEB was responsible for conceptualization, formal analysis, supervision, validation, investigation, visualization, methodology, writing of the original draft, and review and editing. MDR was responsible for validation, methodology, and review. MDP was responsible for validation, methodology, and review. AME was responsible for methodology. ACH was responsible for validation and methodology. PIMC was responsible for visualizations. PK was responsible for data curation. JS was responsible for resources. TLF was responsible for resources. ESC was responsible for data curation. MDG was responsible for resources and review. CM was responsible for conceptualization, supervision, methodology, and review and editing. MPM was responsible for conceptualization, resources, supervision, funding acquisition, investigation, methodology, project administration, and review and editing. CAL was responsible for conceptualization, resources, supervision, funding acquisition, investigation, methodology, project administration, and review and editing.

## Supplementary Material

Supplemental data

Supplemental data 1

Supplemental data 2

Supplemental data 3

Unedited blot and gel images

Supporting data values

## Version 1 09/24/2024

Electronic publication
