# Supplementary material for: Metabolic landscape of the healthy pancreas and pancreatic tumor microenvironment
Source: JCI Insight. 2024 Aug 13;9(18):e180114. doi: 10.1172/jci.insight.180114 (PMC11457849; doi:10.1172/jci.insight.180114)
Supplement: Supplemental data [file jciinsight-9-180114-s077.pdf]

Supplemental Figure1

A

Healthy

Cell Type	Cell Number
Epithelial	7060
CD8	1335
CD4	1244
Granulocyte	349
Acinar	17450
Macrophage	5844
Fibroblast	2405
Endothelial	3911
B-Cells	1715
Pericytes	1165
Mast	159
Cycling	639
Plasma	90
NK	89
Endocrine	529
Dendritic	0
Neural	35

Tumor

Cell Type	Cell Number
Epithelial	9584
CD8	3813
CD4	5074
Granulocyte	4859
Acinar	1290
Macrophage	9085
Fibroblast	2434
Endothelial	396
B-Cells	1209
Pericytes	813
Mast	1682
Cycling	1005
Plasma	1476
NK	1089
Endocrine	18
Dendritic	127
Neural	43

B

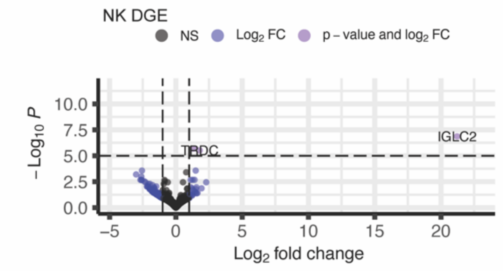

C

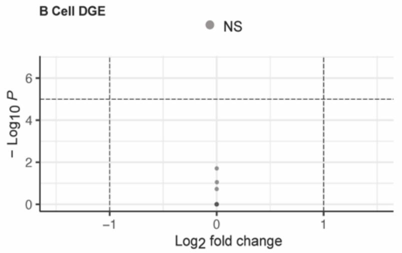

D.

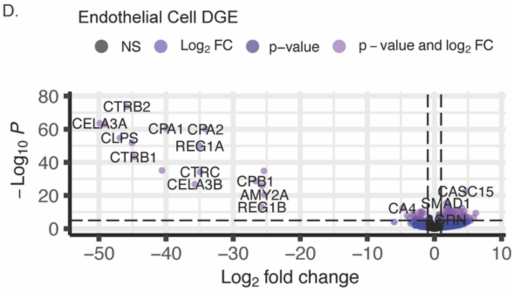

E.

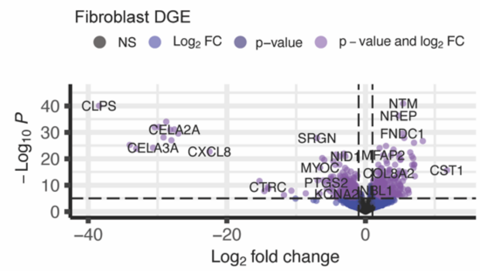

F.

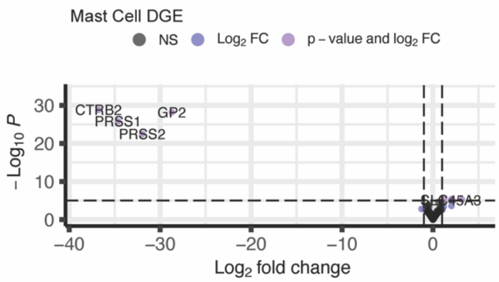

G.

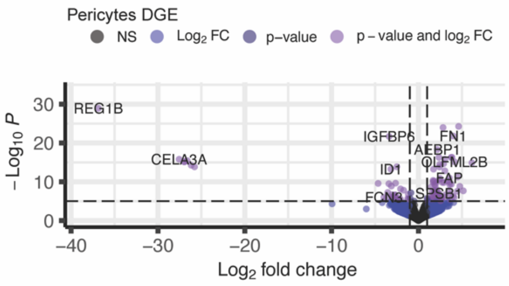

H.

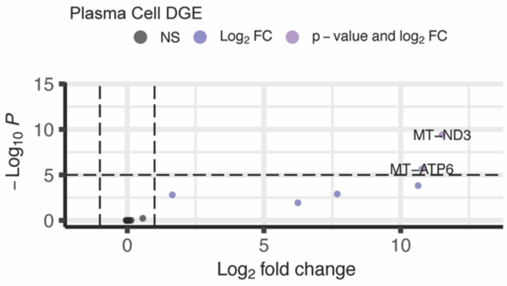

S2: (**A**) GSEA Enrichment plot of oxidative phosphorylation signature significantly increased in cancer cells, with corresponding NES and adjusted p-value.

Supplemental Figure 2

A.

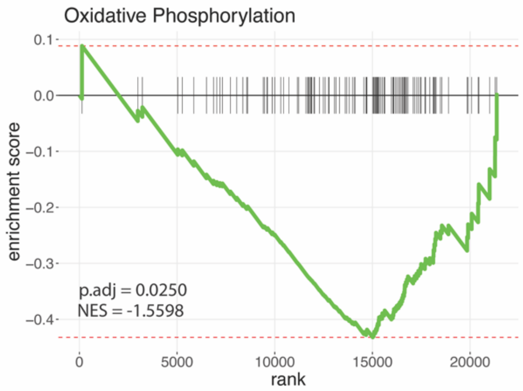

S2: (**A**) GSEA Enrichment plot of oxidative phosphorylation signature significantly increased in cancer cells, with corresponding NES and adjusted p-value.

Supplemental Figure 3

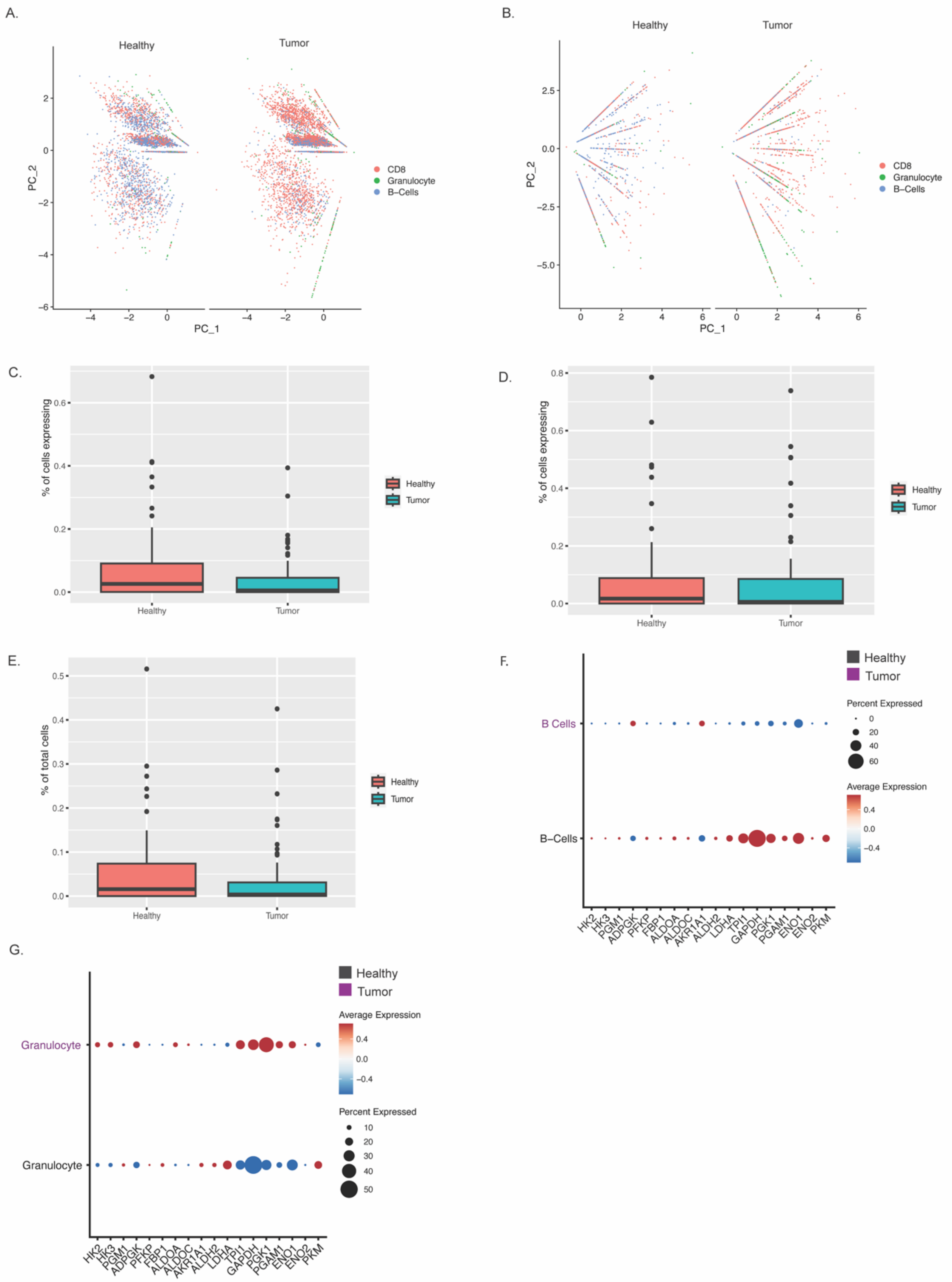

S3: (**A**) Principal Component Analysis (PCA) visualization based on the average expression and percent of cells expressing genes related to complex IV in B cells, granulocytes, and CD8+ T cells in healthy human and PDA samples. (**B**) PCA visualization based on the average expression and percent of cells expressing SDH/A/B/C/D in B cells, granulocytes, and CD8+ T cells in healthy human and PDA samples. (**C-E**) Box plots of reads corresponding to glycolytic genes in healthy and tumor conditions for B cells, CD8+ T cells, and granulocytes. (**F-G**) Dot Plot visualization of glycolytic genes, displaying average expression and percent expressed in B cells and granulocytes, respectively, in tumor (purple) and healthy pancreas tissue (black).

Supplemental Figure 4

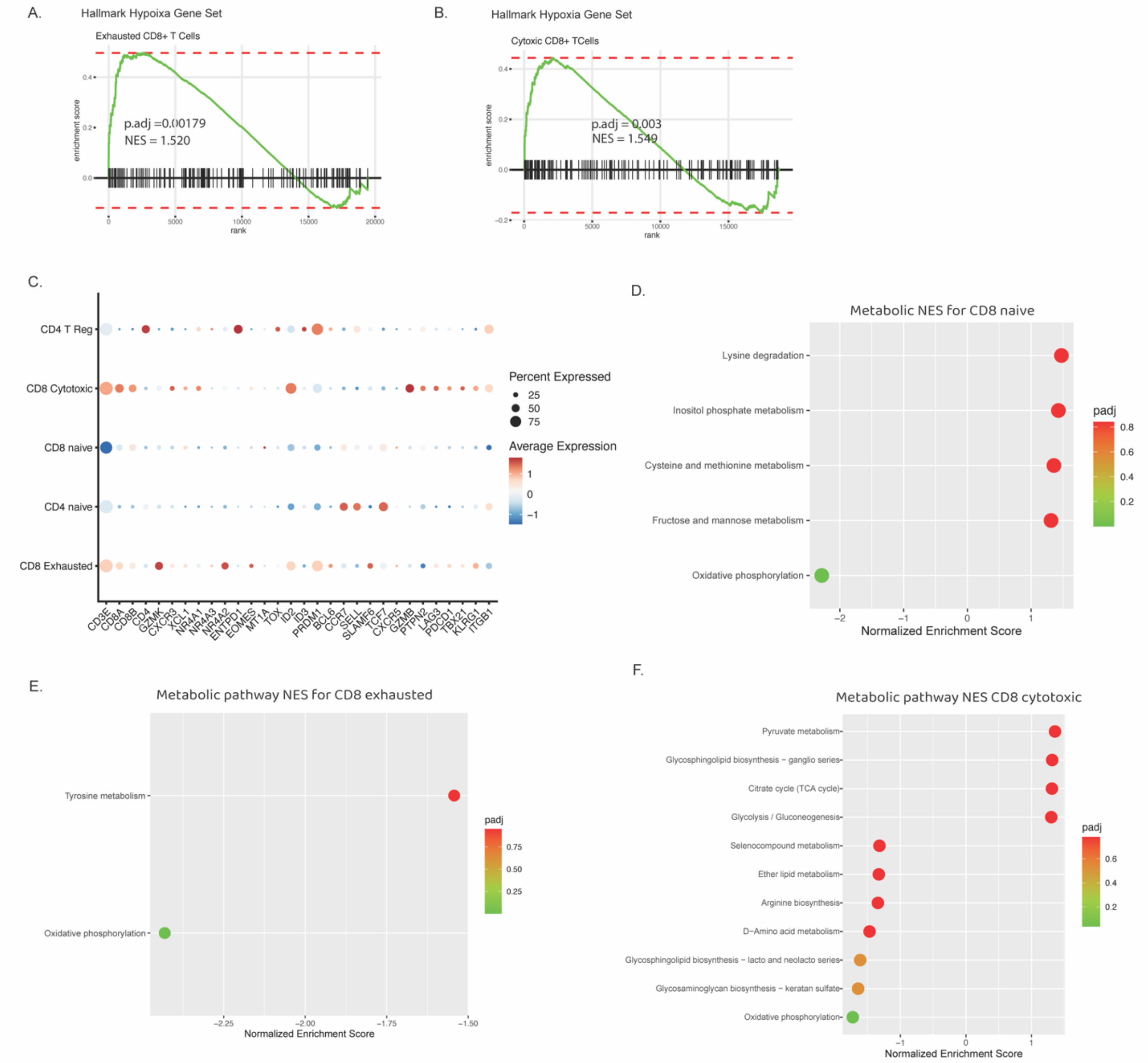

S4: (**A-B)** GSEA Enrichment plots demonstrating hypoxia is significantly downregulated in exhausted and cytotoxic CD8+ T cells in pancreatic cancer tissue compared to healthy counterpart. (**C)**Dot plot visualization of marker expression utilized to subset T cell populations. (**D-F**) GSEA results for CD8 naïve, exhausted, and cytotoxic T cells from PDA samples compared to healthy samples, with corresponding normalized enrichment scores (NES) and adjusted p-values.

Supplemental Figure 5

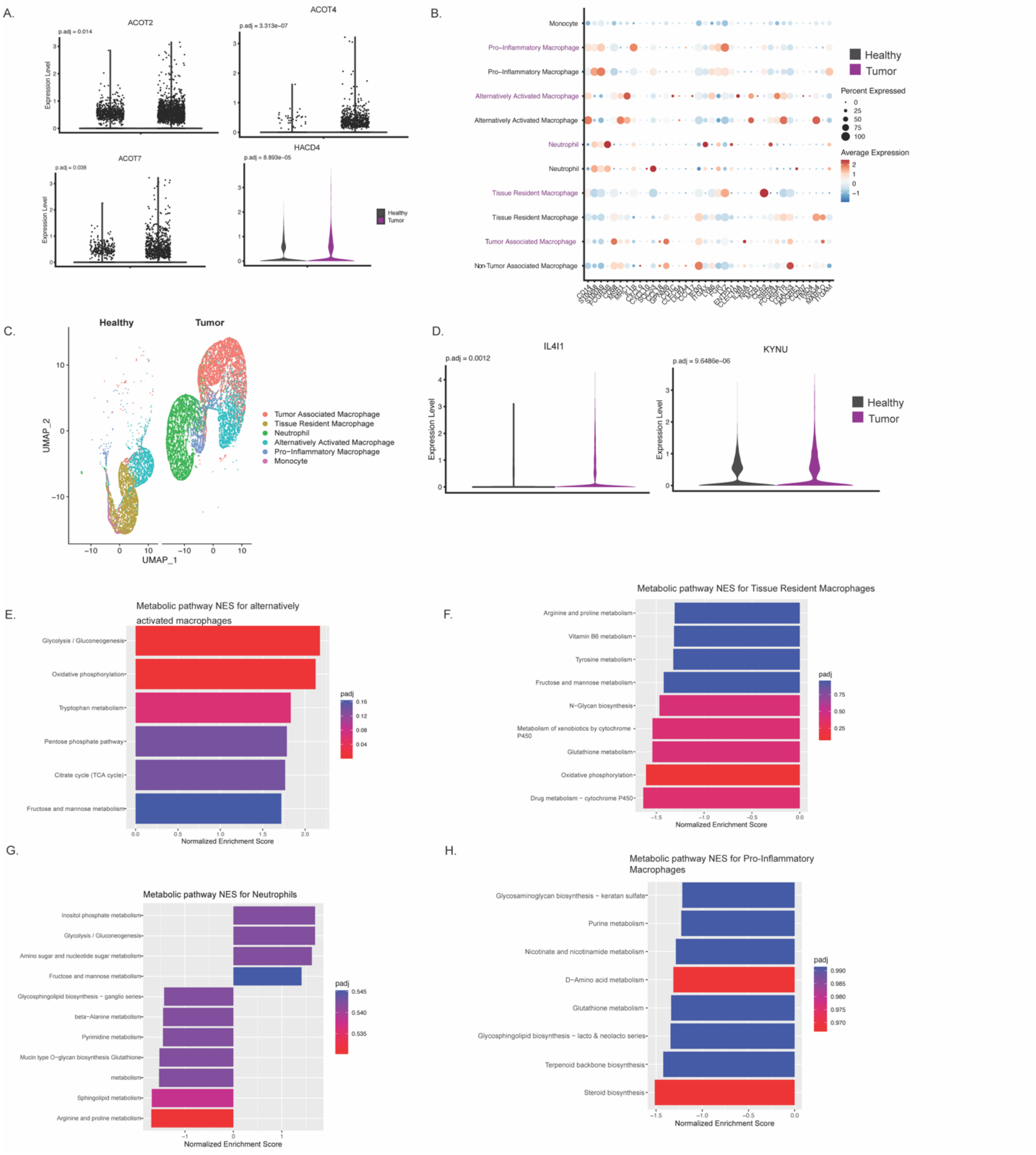

S5: (**A**)Violin plots of genes involved in unsaturated fatty acid synthesis, with adjusted p-values for significantly differentially expressed genes. (**B**) Dot Pot of markers used to identify sub populations of myeloid cells. (**C**) Uniform Manifold Approximation and Projection (UMAP) of myeloid populations in the healthy and tumor conditions. (**D**) Violin plots of differentially genes involved in tryptophan metabolism. **E,** GSEA results for alternatively activated macrophages. (**F**) GSEA results for tissue resident macrophages. (**G**) GSEA analysis for neutrophils. (**H**) GSEA results for pro-inflammatory macrophages. All GSEA results contain corresponding normalized enrichment scores (NES) and adjusted p-values.

Supplementary Figure Legends

S1: (**A)** Table with number of cells per population in the healthy and tumor samples. (**B-H)** Volcano plots of differential gene expression by cell type. Genes that are significantly up- (top-right) and down- regulated (top-left) in tumor versus heathy and the gene symbols are included for representative differentially expressed genes.

S2: (**A)** GSEA Enrichment plot of oxidative phosphorylation signature significantly increased in cancer cells, with corresponding NES and adjusted p-value.

S3: (**A)** Principal Component Analysis (PCA) visualization based on the average expression and percent of cells expressing genes related to complex IV in B cells, granulocytes, and CD8+ T cells in healthy human and PDA samples. (**B)** PCA visualization based on the average expression and percent of cells expressing SDH/A/B/C/D in B cells, granulocytes, and CD8+ T cells in healthy human and PDA samples. (**C-E)** Box plots of reads corresponding to glycolytic genes in healthy and tumor conditions for B cells, CD8+ T cells, and granulocytes. (**F-G)** Dot Plot visualization of glycolytic genes, displaying average expression and percent expressed in B cells and granulocytes, respectively, in tumor (purple) and healthy pancreas tissue (black).

S4: (**A-B)** GSEA Enrichment plots demonstrating hypoxia is significantly downregulated in exhausted and cytotoxic CD8+ T cells in pancreatic cancer tissue compared to healthy counterpart. (**C)**Dot plot visualization of marker expression utilized to subset T cell populations. (**D-F**) GSEA results for CD8 naïve, exhausted, and cytotoxic T cells from PDA samples compared to healthy samples, with corresponding normalized enrichment scores (NES) and adjusted p-values.

S5: (**A)**Violin plots of genes involved in unsaturated fatty acid synthesis, with adjusted p-values for significantly differentially expressed genes. (**B)** Dot Pot of markers used to identify sub populations of myeloid cells. (**C**) Uniform Manifold Approximation and Projection (UMAP) of myeloid populations in the healthy and tumor conditions. (**D)** Violin plots of differentially genes involved in tryptophan metabolism. **E,** GSEA results for alternatively activated macrophages. (**F)** GSEA results for tissue resident macrophages. (**G)** GSEA analysis for neutrophils. (**H)** GSEA results for pro-inflammatory macrophages. All GSEA results contain corresponding normalized enrichment scores (NES) and adjusted p-values.
