# Supplementary figures and images for: Metabolic landscape of the healthy pancreas and pancreatic tumor microenvironment
Source: JCI Insight. 2024 Aug 13;9(18):e180114. doi: 10.1172/jci.insight.180114 (PMC11457849; doi:10.1172/jci.insight.180114)

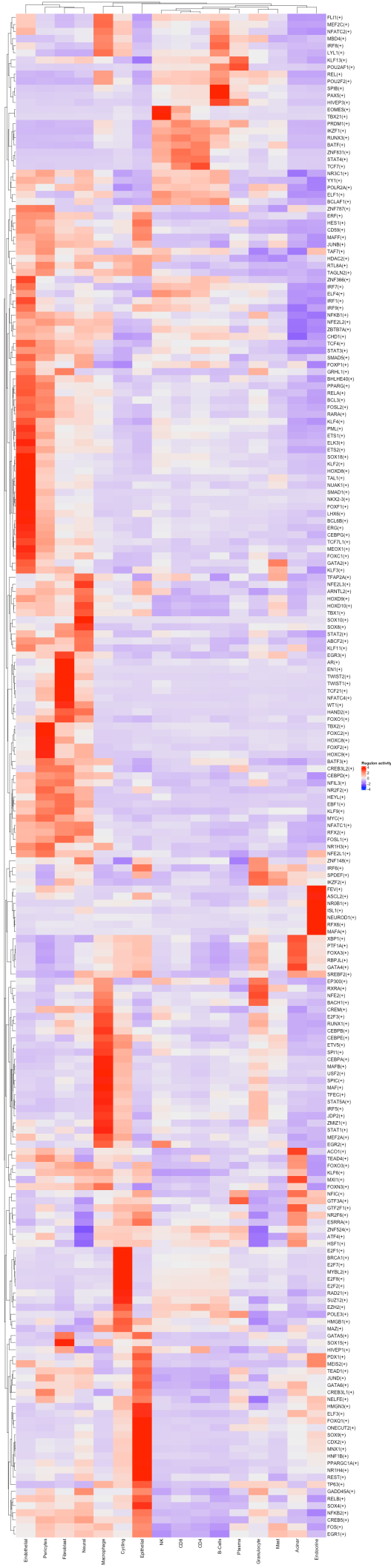

Supplement: Supplemental data 1 [file jciinsight-9-180114-s078.pdf]

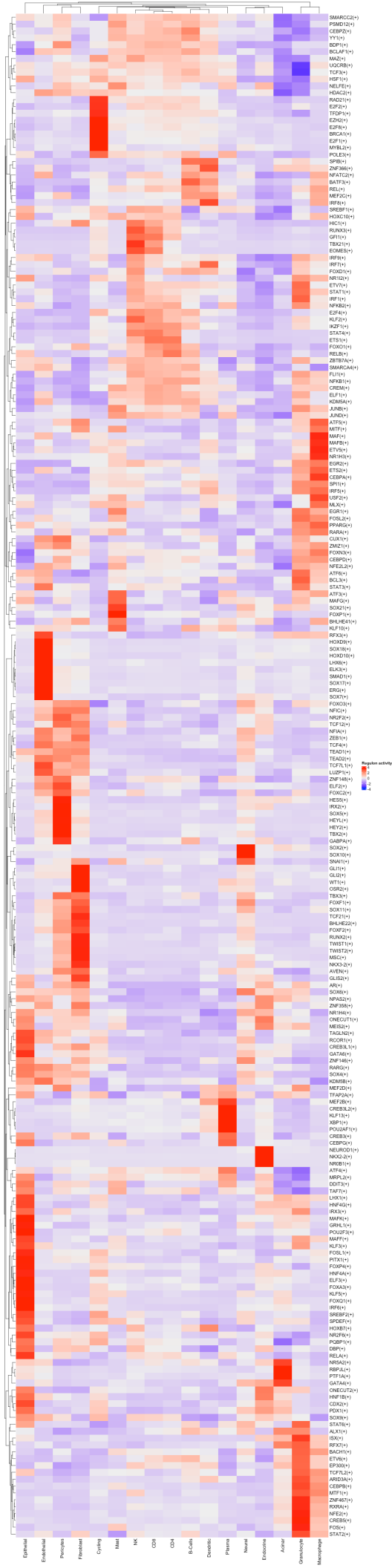

Supplement: Supplemental data 2 [file jciinsight-9-180114-s079.pdf]

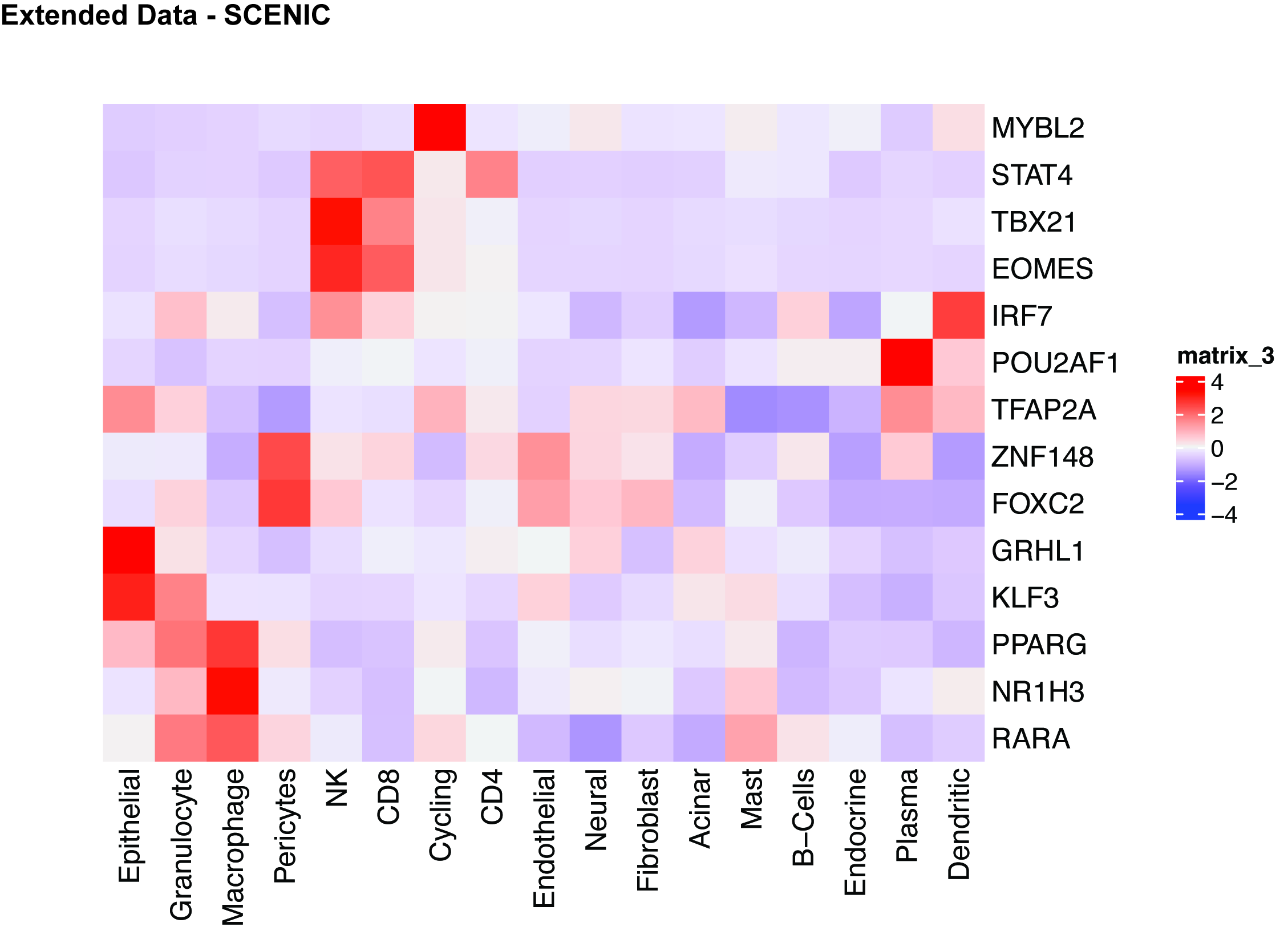

Supplement: Supplemental data 3 [file jciinsight-9-180114-s080.tif]

Full unedited blot for **Figure 5D**

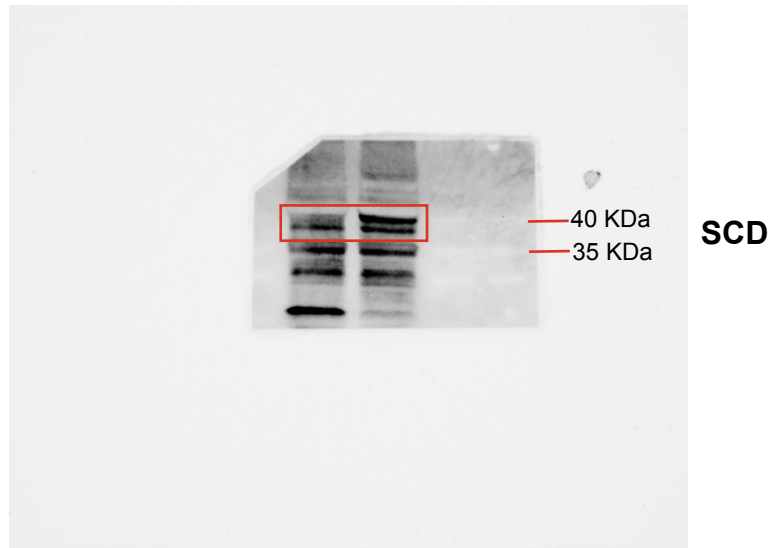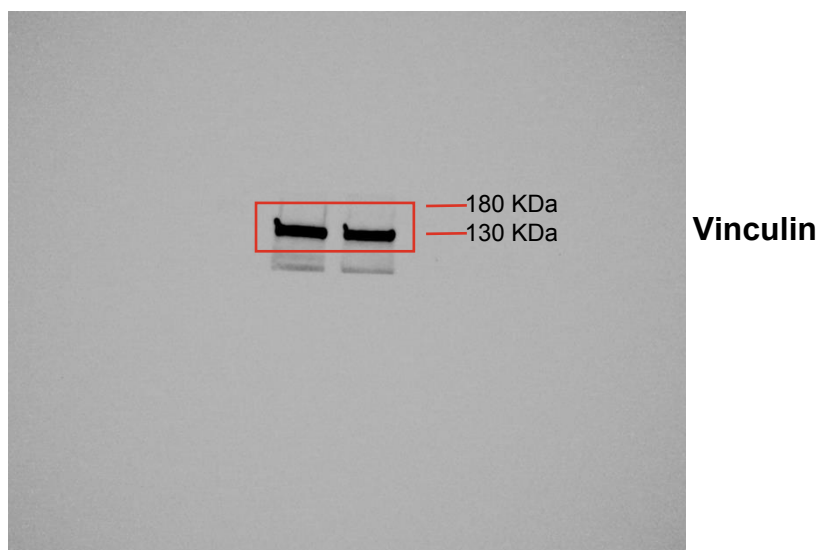

Full unedited blot for **Figure 6D**

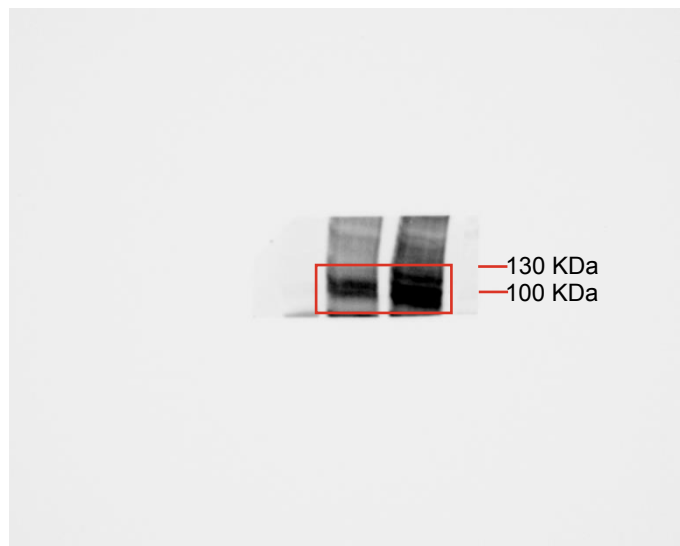

**ABCG1**

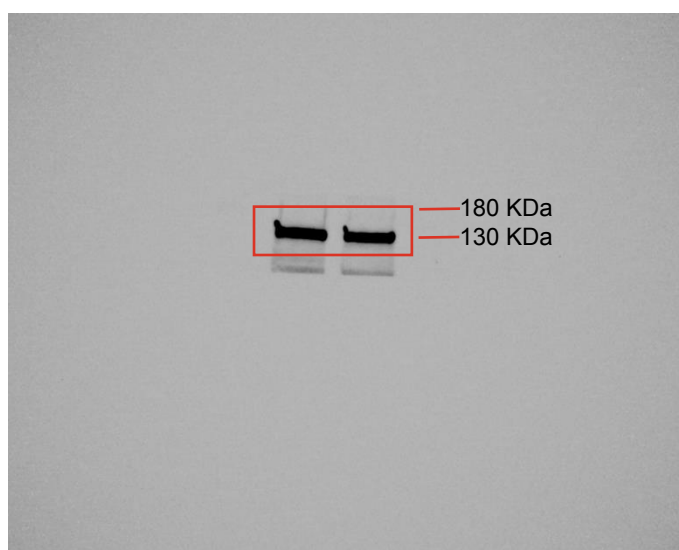

**Vinculin**

Supplement: Unedited blot and gel images [file jciinsight-9-180114-s081.pdf]
